# Association between onset age and mortality gradients in advanced cardiovascular–kidney–metabolic syndrome

**DOI:** 10.3389/fendo.2025.1648083

**Published:** 2025-09-03

**Authors:** Jinjin Dai, Chuanchang Wu, Shihua Liu, Shuohua Chen, Xiaodan Hong, Pengyue Zhang, Liufu Cui, Shouling Wu, Zhenhua Zhang

**Affiliations:** ^1^ Department of Infectious Diseases, The Second Affiliated Hospital of Anhui Medical University, Hefei, Anhui, China; ^2^ Department of Infectious Diseases, Suzhou Hospital of Anhui Medical University, Suzhou, Anhui, China; ^3^ Department of Neurology, Suzhou Hospital of Anhui Medical University, Suzhou, Anhui, China; ^4^ Department of Cardiology, Kailuan General Hospital, Tangshan, China; ^5^ Department of Endocrinology, Kailuan General Hospital, Tangshan, China

**Keywords:** cardiovascular-kidney-metabolic syndrome, onset age, mortality gradient, absolute risk burden, stratified prevention

## Abstract

**Introduction:**

Advanced cardiovascular–kidney–metabolic (A-CKM) syndrome portends severe prognosis, but how onset age affects mortality risk remains unquantified.

**Methods:**

This study analyzed 179,328 participants from the Kailuan cohort in Tangshan, China (2006–2022). Using weighted Cox models and stratified analyses, we assessed the association of age at onset with all-cause mortality risk.

**Results:**

Among 17,283 incident A-CKM cases matched to age-stratified controls, early-onset patients (<45 y) had the highest relative mortality risk (HR = 3.35), which was amplified by smoking (HR = 5.27) and inflammation (hsCRP≥3mg/L: HR = 10.15); midlife onset (45– 54 y) represented the optimal prevention window (NNT = 15), yet with extreme female vulnerability (Stage 4 HR=14.25 vs. male HR=2.54); late-adulthood onset (55–64y) incurred peak absolute burden (ΔRate +8.61/1000PY), while elderly cases (≥65 y) had an attenuated attributable impact despite higher mortality (33.95 vs. 2.48/1000 PY).

**Discussion:**

These findings support an age-stratified management framework: core age phased priorities (risk containment <45 y, preventive interception 45 – 54 y, complication management 55 – 64 y, and renoprotective optimization ≥65 y) augmented by sex-specific refinements—aggressive inflammation control in young men and intensified midlife monitoring for women—resolving the efficiency-burden paradox through calibrated implementation.

**Clinical trial registration:**

https://www.chictr.org.cn/showproj.html?proj=8050, identifier ChiCTR-TNRC-11001489.

## 1 Introduction

Cardiovascular disease (CVD), chronic kidney disease (CKD), and metabolic syndrome (MetS) share overlapping risk factors and exhibit pathophysiological interactions that synergistically amplify adverse clinical outcomes beyond simple additive effects (1). To systematically characterize this multisystem disease cluster and establish an integrated management framework, the American Heart Association proposed cardiovascular–kidney–metabolic syndrome (CKM) (2), which affects more than 30% of adults worldwide (3). CKM highlights the continuous and progressive characteristics of multiple organ dysfunction and presents a qualitative staging system, with nonadvanced stages (Stage 0, 1, or 2) and advanced stages (Stage 3 or 4). Advancements in CKM staging enable more accurate identification of high-risk individuals (4). Notably, advanced CKM syndrome (A-CKM) patients exhibit a significantly elevated risk of all-cause mortality (5). Specifically, a 15-year follow-up study revealed that the all-cause mortality rate among these patients reached 37.9 per 1000 person-years (6).

Aging is a well-established pivotal risk factor for CVD (7, 8). However, our prior cohort analysis revealed a paradoxical reversal of mortality risk in A-CKM. Specifically, adults aged <60 years faced a 3.4-fold higher mortality risk compared to their older counterparts (adjusted HR 8.07 vs. 3.44) (6). Elucidating this phenomenon requires analysis through biological aging metrics. BioAge/PhenoAge Dissociation: PhenoAge demonstrates superior predictive capacity for advanced CKM stages versus BioAge, evidenced by significantly lower nonlinearity (9). Epigenetic Acceleration: Each 5-unit increment in GrimAge acceleration confers 50% excess mortality, mechanistically decoupling biological decline from chronological aging (10). The PREVENT model in the U.S. incorporated temporal dimensions to assess risk, verifying that the age of onset and duration of illness are risk factors meriting inclusion in future updates of guidelines for CKM (11). However, age-stratified analyses of CKM-associated mortality risk remain rare. Exploring age-of-onset-associated risk differences is crucial for enhancing risk stratification and customizing potential intervention strategies.

We conducted a population-based prospective community cohort study in China. While early-stage CKM demonstrates limited predictive validity for mortality (6), our study enhances methodological rigor by incorporating A-CKM criteria to assess all-cause mortality risk. We aimed to explore the association between onset age and all-cause mortality in patients with A-CKM and investigate differences in risk factors according to age of onset. This work provides a basis for optimizing early interventions and developing age-stratified CKM management strategies.

## 2 Methods

### 2.1 Study design and participants

The Kailuan Study (registration number: ChiCTRTNRC-11,001,489) is a prospective cohort study of the investigation of and interventions for CVD and related risk factors that is being conducted with community-based participants. The participants in this study were employees and retirees of the Kailuan Group, a large coal mining enterprise in the Tangshan region. Detailed descriptions of the study design and procedures have been published elsewhere (12). Briefly, from June 2006 to December 2017, a total of 179,328 participants aged 18 to 98 years were enrolled. These participants underwent biennial questionnaire surveys, clinical examinations, and laboratory tests at 11 hospitals affiliated with the Kailuan Group. Additionally, the occurrence of chronic diseases and mortality events was recorded annually. All the involved participants were followed up until December 31, 2022. The study was conducted in strict accordance with the ethical principles outlined in the Declaration of Helsinki and was approved by the Ethics Committee of Kailuan General Hospital (approval number: 200605). Written informed consent was obtained from all participants prior to their inclusion in the study.

In the present study, we excluded participants with missing data for body mass index (BMI), waist circumference (WC), fasting blood glucose (FBG), systolic blood pressure (SBP), diastolic blood pressure (DBP), triglycerides (TG), total cholesterol (TC), low-density lipoprotein cholesterol (LDL-C), high-density lipoprotein cholesterol (HDL-C), serum creatinine, or proteinuria, resulting in the inclusion of 165,701 participants who met the study criteria. The date of new-onset A-CKM was defined as the date of the health examination at which A-CKM was first diagnosed.

New-onset A-CKM was prospectively ascertained through serial biennial examinations (2006 – 2017). Cases required ≥2 examinations with confirmed disease-free status at baseline (Exam_0_) and incident diagnosis at subsequent examination (Exam_1_). Onset age was operationally defined as the date of the first diagnostic examination (Exam_1_). This approach minimizes misclassification versus midpoint imputation, particularly in sparse data contexts (Example: Participant disease-free in 2012 and diagnosed in 2014→Onset = 2014). Matched case–control pairs were constructed with calendar-year synchronization to eliminate immortal time bias. Each case was matched to a control initiating follow-up at their health examination during the same calendar year. Controls were randomly selected participants from the same examination cycle, matched by sex, ± 1-year age range, and the absence of A-CKM at all prior examinations. Both cohorts began follow-up at their respective Exam_1_ dates, generating 17,283 case–control pairs with temporally aligned observations. This methodology was uniformly applied to CKM Stages 3/4/4a/4b. Age thresholds (<45, 45 – 54, 55 – 64, and ≥65 years) were defined by: (1) Guideline screening windows (ACC/AHA): (2) Chinese population transition peaks (45 - 54y: 184% A-CKM prevalence increase vs. <45y) (13), (3) Biological decline inflection (≥65y renal decline onset + 56.6% disease burden) (14), and (4) Statistical power assurance (n≥1,000/stratum). All incident cases were confirmed through 57 predefined diagnostic trajectories (**Supplementary Table S1**), characterized by three principal patterns: immediate (0→1) transitions requiring disease-free status within 2 years prediagnosis; delayed (0→NA→1) transitions allowing single missed examinations with verified disease-free status at last attendance; and multistep (0→0→1) transitions demanding consecutive disease-free records. To ensure diagnostic precision, cases were required to demonstrate documented disease-free status at their immediately preceding examination, whereas matched controls exhibited persistent absence of A-CKM at all study examinations throughout the observation period.

### 2.2 Data collection and variable definitions

The detailed methods of the epidemiological survey and anthropometric measurements have been previously described (15, 16). All examinations were conducted in climate-controlled rooms (22 ± 2°C, 40 - 60% relative humidity) using standardized protocols. Certified physicians performed measurements: blood pressure was recorded with calibrated mercury sphygmomanometers after ≥15 minutes of seated rest (right arm, mean of ≥2 readings with remeasurement if >5 mmHg difference); weight and height were measured to 0.1 kg/cm precision using SECA scales (light clothing, no shoes); waist circumference was determined at the midpoint between the iliac crest and lowest rib margin.

Venous blood samples collected after ≥8-hour fasting were transported to the central laboratory within ≤30 minutes using EDTA-containing vacuum tubes. Serum separation was achieved by centrifugation at 3000 rpm for 10 minutes (Eppendorf 5810R, Germany), followed by analysis on Hitachi 747 and Roche Cobas c501 systems. Comprehensive methodological specifications including analytical principles, detection ranges, and precision data (intra/inter-assay CV) are provided in **Supplementary Table S2**. All laboratory procedures adhered to ISO 15189:2012 requirements.

Variable definitions were established as follows: BMI was derived from weight (kg) divided by height squared (m²). Hypertension diagnosis required SBP≥140 mmHg and/or DBP≥90 mmHg, self-reported clinical history, or use of antihypertensive medications within the preceding two weeks (17). Diabetes mellitus status was determined by FBG ≥7.0 mmol/L (quantified through hexokinase/G6PDH enzymatic assay; see **Supplementary Table S2** for validation data), clinically documented diagnosis, or hypoglycemic agent use (18). Dyslipidemia classification incorporated lipid biomarkers: TC ≥5.2 mmol/L, LDL-C ≥3.4 mmol/L, HDL-C <1.0 mmol/L, or triglycerides ≥1.7 mmol/L (all measured via methods detailed in **Supplementary Table S2**), alongside self-reported history or lipid-lowering therapy (19). eGFR was calculated via CKD-EPI equation (20). Midstream morning urine specimens used DIRUI N - 600 analyzers. CKD risk was stratified per 2012 KDIGO guidelines (21) using IDMS-standardized serum creatinine measurements. CVD encompassed physician-adjudicated composite endpoints including myocardial infarction, atrial fibrillation, stroke, and heart failure, verified through structured electronic health record extraction. MetS required ≥3 of the following (22): waist circumference ≥90 cm (men) or ≥80 cm (women); HDL-cholesterol <1.04 mmol/L (men) or <1.29 mmol/L (women) (method: **Supplementary Table S2**); triglycerides ≥1.7 mmol/L (method: **Supplementary Table S2**); blood pressure ≥130/80 mmHg or antihypertensive use; fasting blood glucose ≥5.6 mmol/L (method: **Supplementary Table S2**). Concurrently, structured epidemiological questionnaires documented lifestyle factors including current smoking (≥1 cigarette/day), regular alcohol consumption (≥1 alcoholic beverage/day during the previous year), and physical activity engagement (≥30 minutes/session, ≥3 times weekly).

### 2.3 Definitions of CKM syndrome stages

In accordance with the scientific statement from the AHA, CKM is classified into distinct stages (Stage 0 to Stage 4) (22). Stage 0 is characterized by the absence of CKM risk factors, including normal BMI and WC, normoglycemia, normotension, normal lipid profiles, and no evidence of CKD or subclinical or clinical CVD. Stage 1 is defined as the presence of at least one of the following: excess weight (BMI ≥23 kg/m^2^), abdominal obesity (WC ≥80 cm (women) and ≥90 cm (men)), or dysfunctional adipose tissue, clinically manifested as impaired glucose tolerance or prediabetes (5.6 mmol/L≤FBG ≤6.9 mmol/L), in the absence of additional metabolic risk factors or CKD. Stage 2 is defined by the presence of metabolic risk factors, including hypertriglyceridemia (TG ≥1.5 mmol/L), dyslipidemia, hypertension, MetS, or diabetes, as well as moderate-to-high-risk CKD, or a combination of these conditions. Stage 3 indicates subclinical CVD, defined indirectly using risk equivalents due to the lack of direct indicators, including very high-risk CKD and a predicted 10-year CVD risk ≥20% using the Prediction for Atherosclerotic Cardiovascular Disease Risk in China (China-PAR) model (23). Stage 4 represents clinical CVD and is subdivided into two categories: Stage 4a (clinical CVD without kidney failure) and Stage 4b (clinical CVD with concurrent kidney failure). Participants were stratified into non-advanced (stages 0 - 2) or advanced CKM (stages 3 - 4), encompassing individuals with CVD or those at high risk of development (**Supplementary Table S3**).

### 2.4 Outcomes

All-cause mortality was defined as death from any cause during the follow-up period. This outcome was verified annually by professional physicians through systematic review of official death certificates sourced from provincial vital statistics agencies (24). Participants were followed from the baseline assessment date until the occurrence of death or December 31, 2022, whichever occurred first.

### 2.5 Statistical analysis

Case subjects with new-onset A-CKM and matched controls were stratified into four age-at-onset groups (<45, 45 - 54, 55 - 64, ≥65 years). Normality for all continuous variables was formally assessed using the Kolmogorov-Smirnov test (α=0.05). Normally distributed variables are presented as mean ± standard deviation with group comparisons via ANOVA; non-normal variables as median (IQR) analyzed by Kruskal-Wallis test. Categorical variables are reported as frequencies (%) with chi-square testing. Incidence density was calculated per 1,000 person-years. Cox proportional hazards models (age-scaled) estimated mortality hazard ratios (95% CI), adjusted for smoking, alcohol intake, physical activity, education, occupation, and hs-CRP. Equivalent frameworks evaluated CKM Stages 3/4/4a/4b.

We conducted additional subgroup analyses which stratified participants by baseline smoking status, alcohol consumption status, hypertension status, diabetes status, dyslipidemia status, MetS status, hs-CRP level, and HDL-C level. Exploratory analyses further examined the mortality impact of individual Stage 3 CKM components.

Sensitivity assessments excluded (1) events occurring within the first year of follow-up to mitigate potential reverse causality and (2) participants reporting regular physical activity and using medications. The population-attributable fraction (PAF) was calculated using the Miettinen formula: PAF = p_c_ (1 - 1/RR), where RR and p_c_ denote the relative risk and the incidence of exposure among cases, respectively. The number needed to treat (NNT) was calculated as NNT = 1/[(ARD/1000)×T], with absolute risk difference (ARD) = (Event rate_A-CKM_−Event rate_Control_) per 1,000 person-years and T = 10-year time scale. This represents the number of patients who needed treatment for more than 10 years to prevent one adverse event. 95% confidence intervals (CIs) were computed using Fieller’s method to account for ratio metric nonnormality, where CI = 1000/[(ARD ± 1.96×SE) ×T] and the standard error derived from the Poisson variance was SE(ARD) =1000×, where E=events and PY=person-year. Sensitivity analyses with nonparametric bootstrapping (1,000 replicates) confirmed CI stability (deviation <5%). Statistical analyses were performed using SAS 9.4 (SAS Institute) and R 4.2.0 (R Project for Statistical Computing). Two-sided *P* < 0.05 was considered to indicate statistical significance.

## 3 Results

### 3.1 Baseline characteristics

The baseline characteristics of the 17,283 A-CKM case–control pairs of participants are presented in **Table 1**. Among them, 89.40% were male, and the mean onset age of A-CKM was 61.29 ± 12.14 years. Compared with controls, A-CKM cases exhibited significantly higher proportions of current drinkers and physical activity; notable metabolic disturbances included elevated WC, BMI, SBP, DBP, FBG, LDL-C, TG, TC, and hs-CRP. The prevalence of MetS, hypertension, diabetes, dyslipidemia, and family history of CVD was significantly higher among A-CKM cases. Conversely, A-CKM cases demonstrated lower eGFR and HDL-C levels, with fewer participants engaged in mental work.

**Table 1:** Baseline characteristics of patients with new-onset advanced cardiovascular-kidney-metabolic syndrome (A-CKM) and matched controls.

Characteristics	Control subjects	New-onset A-CKM	*P* value	A-CKM onset age (years)				*P* for trend
				<45	45-54	55-64	≥65	
No. of participants	17283	17283	/	1336	2319	6303	7325	/
Age, years	61.29 ± 12.14	61.29 ± 12.14	–	36.36 ± 6.40	51.01 ± 2.83	60.52 ± 2.77	70.70 ± 4.27	–
Men, n (%)	15451 (89.40)	15451 (89.40)	–	1189 (89.00)	2020 (87.11)	5657 (89.75)	6585 (89.90)	0.001
Current drinkers, n (%)	4778 (27.65)	4992 (28.88)	0.011	701 (52.47)	990 (42.69)	1853 (29.40)	1448 (19.77)	<0.001
Current smokers, n (%)	5437 (31.46)	5322 (30.79)	0.182	661 (49.48)	1099 (47.39)	2177 (34.54)	1385 (18.91)	<0.001
Physical activity, n (%)	3033 (17.55)	3478 (20.12)	<0.001	124 (9.28)	355 (15.31)	1347 (21.37)	1652 (22.55)	<0.001
High school or above, n (%)	2584 (14.95)	2510 (14.52)	0.262	631 (47.23)	513 (22.12)	620 (9.84)	746 (10.18)	<0.001
Mental work, n (%)	2520 (14.58)	2408 (13.93)	0.006	246 (18.41)	284 (12.25)	712 (11.30)	1166 (15.92)	<0.001
WC, cm	87.47 ± 9.39	88.97 ± 10.34	<0.001	87.36 ± 10.41	89.64 ± 10.09	89.53 ± 10.09	88.58 ± 10.56	<0.001
BMI, kg/m^2^	24.64 ± 3.27	25.62 ± 3.38	<0.001	25.30 ± 3.81	25.86 ± 3.41	25.84 ± 3.25	25.42 ± 3.38	<0.001
SBP, mmHg	134.50 ± 17.87	152.52 ± 24.50	<0.001	130.18 ± 21.21	145.19 ± 27.35	156.24 ± 24.96	155.72 ± 20.77	<0.001
DBP, mmHg	82.59 ± 10.44	88.80 ± 13.27	<0.001	82.46 ± 13.32	90.88 ± 14.87	91.40 ± 13.25	87.07 ± 12.01	<0.001
FBG, mmol/L	5.68 ± 1.78	6.61 ± 2.78	<0.001	5.61 ± 2.98	6.37 ± 2.69	6.69 ± 3.04	6.55 ± 2.47	<0.001
LDL-C, mmol/L	2.74 ± 1.23	2.87 ± 1.13	<0.001	2.95 ± 1.47	2.80 ± 1.52	2.89 ± 1.08	2.87 ± 0.93	<0.001
HDL-C, mmol/L	1.48 ± 0.57	1.34 ± 0.52	<0.001	1.50 ± 1.03	1.38 ± 0.53	1.31 ± 0.46	1.33 ± 0.41	<0.001
TG, mmol/L	1.17 (0.83, 1.77)	1.44 (1.01, 2.21)	<0.001	1.43 (0.99, 2.36)	1.55 (1.07, 2.62)	1.55 (1.08, 2.40)	1.34 (0.96, 1.97)	<0.001
TC, mmol/L	4.94 ± 1.18	5.16 ± 1.32	<0.001	4.98 ± 1.17	5.16 ± 1.42	5.23 ± 1.40	5.12 ± 1.23	<0.001
hs-CRP, mg/L	1.10 (0.46, 2.60)	1.42 (0.60, 3.10)	<0.001	1.64 (1.10, 2.50)	1.50 (0.70, 3.00)	1.30 (0.57, 2.98)	1.43 (0.56, 3.38)	<0.001
eGFR, mL/min/1.73 m^2^	88.22 ± 19.50	77.43 ± 27.57	<0.001	40.27 ± 37.40	80.15 ± 34.11	83.58 ± 23.99	78.06 ± 19.69	<0.001
Family history of CVD, n (%)	360 (2.08)	491 (2.84)	<0.001	49 (3.67)	152 (6.55)	195 (3.09)	95 (1.30)	<0.001
MetS, n (%)	5725 (33.13)	9333 (54.00)	<0.001	446 (33.38)	1268 (54.68)	3815 (60.53)	3804 (51.93)	<0.001
Hypertension, n (%)	8030 (46.46)	13231 (76.55)	<0.001	399 (29.87)	1535 (66.19)	5116 (81.17)	6181 (84.38)	<0.001
Diabetes, n (%)	1505 (8.71)	5473 (31.67)	<0.001	108 (8.08)	581 (25.05)	2437 (38.66)	2347 (32.04)	<0.001
Dyslipidemia, n (%)	10242 (59.26)	12462 (72.11)	<0.001	827 (61.90)	1721 (74.21)	4812 (76.34)	5102 (69.65)	<0.001
Anti-hypertension drugs, n (%)	1043 (6.03)	3187 (18.44)	<0.001	81 (6.06)	567 (24.45)	1260 (19.99)	1279 (17.46)	<0.001
Hypoglycemic drugs, n (%)	446 (2.58)	1586 (9.18)	<0.001	22 (1.65)	188 (8.11)	703 (11.15)	673 (9.19)	<0.001
Lipid-lowering drugs, n (%)	75 (0.43)	272 (1.57)	<0.001	16 (1.20)	73 (3.15)	110 (1.75)	73 (1.00)	<0.001

Data presented as mean ± standard deviation (SD), median (interquartile range), or number (percentage).

BMI, body mass index; CKD, chronic kidney disease; CVD, cardiovascular disease; DBP, diastolic blood pressure; eGFR, estimated glomerular filtration rate; FBG, fasting blood glucose; HDL-C, high-density lipoprotein cholesterol; hs-CRP, high-sensitivity C-reactive protein; LDL-C, low-density lipoprotein cholesterol; MetS, metabolic syndrome; SBP, systolic blood pressure; TC, total cholesterol; TG, triglycerides; WC, waist circumference.

When stratified by onset age groups (<45, 45 – 54, 55 – 64, and ≥65 years), younger-onset A-CKM participants exhibited distinct phenotypes: higher levels of current smoking and alcohol use, physical inactivity, and elevated HDL-C and hs-CRP concentrations. Compared with older-onset A-CKM patients, younger-onset patients had lower WC, BMI, blood pressure, FBG, and eGFR levels and a lower prevalence of MetS, hypertension, diabetes, and dyslipidemia. Moreover, **Supplementary Tables S4–S7** display the baseline characteristics for Stage 3, 4, 4a, and 4b CKM case–control pairs, respectively.

### 3.2 Associations between A-CKM onset age and all-cause mortality

**Figure 1:**
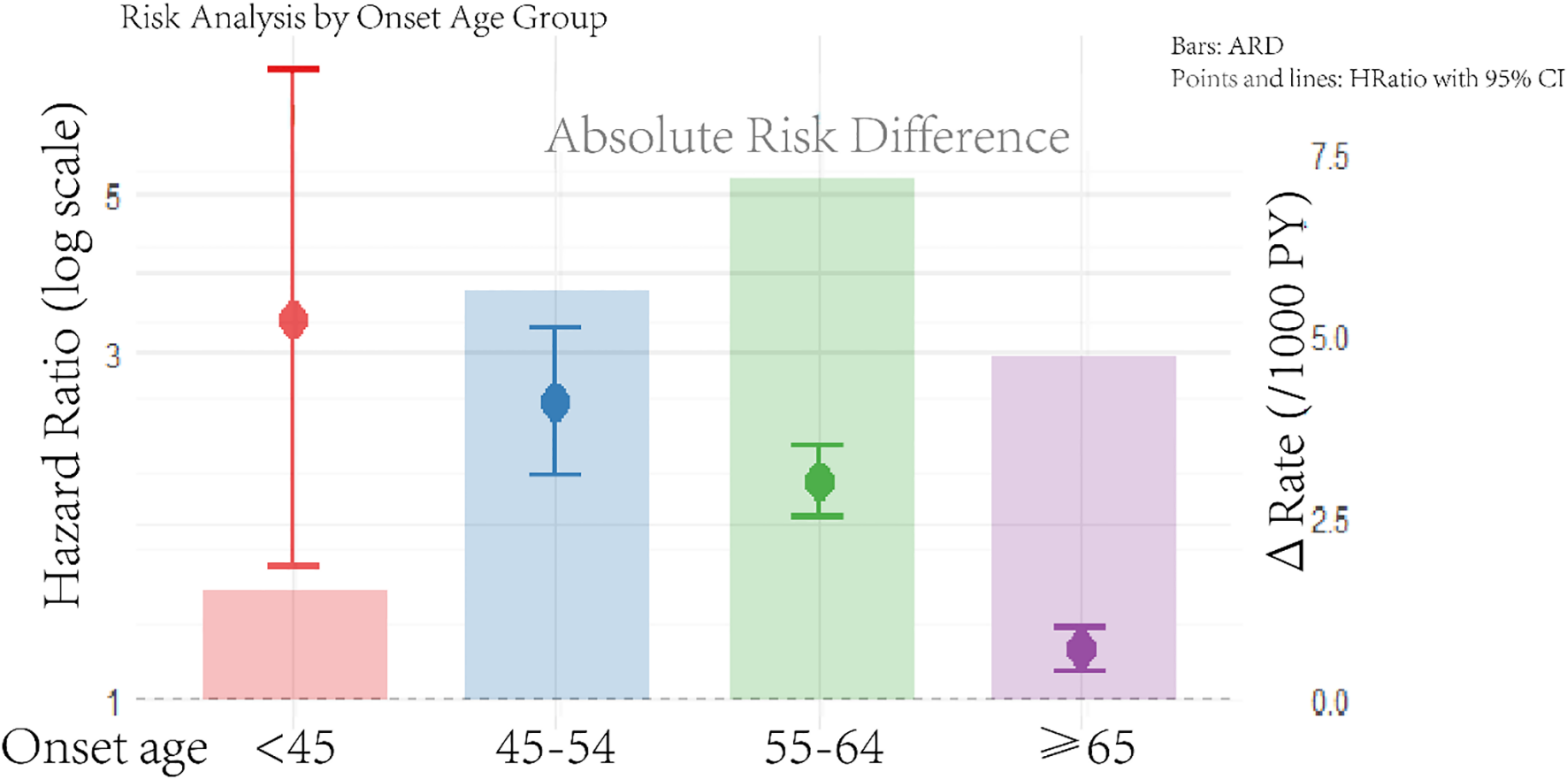
Age-stratified mortality risk in incident advanced CKM syndrome: hazard ratios and absolute risk difference. The rate is per 1,000 person-years. The model was adjusted for smoking status, drinking status, physical activity, education, work type, and high-sensitivity C-reactive protein. Hazard ratios (HR, left axis; logarithmic scale) derived from Cox models; absolute risk differences (ΔRate/1000 PY, right axis; linear scale) calculated via Poisson regression. Reference line: HR = 1.0 (dashed) corresponding to ΔRate=0. The full regression outputs are listed in **Supplementary Table S4**.

During a median follow-up of 8.14 years, we documented 5,547 deaths among case–controls. The age-stratified incidence rates (per 1,000 person-years) and multivariate-adjusted HRs for A-CKM cases versus matched controls are presented in **Figure 1**, which integrates two key dimensions: hazard ratios (forest plot) and absolute risk differences (event rates with ΔRate). After multivariate adjustment, new-onset A-CKM patients had a significantly higher mortality risk across all onset age groups. Consistent with previous findings, the highest relative risk was observed in the youngest cohort (HR, 3.35; 95% CI, 1.52 – 7.38), and the risk decreased with advancing age (45 – 54 years: HR 2.58, 95% CI 2.04 – 3.26; 55 – 64 years: HR 2.00, 95% CI 1.79 – 2.23; ≥65 years: HR 1.17, 95% CI 1.09 – 1.25; *P* for interaction <0.001). Absolute risk analysis revealed a distinct pattern: the greatest excess risk occurred in the 55 – 64-year group (ΔRate +8.61/1,000 person-years), followed by the 45 – 54-year group (ΔRate +6.75). Although this group (55 – 64 years) had a lower relative risk than the <45-year cohort did, it carried the greatest absolute mortality burden. Conversely, the <45-year group had the smallest absolute risk increase (ΔRate +1.79) despite having the highest HR. On the basis of the integrated risk assessment, the 45 – 54-year and 55 – 64-year groups were classified as Priority I (highest intervention urgency), the ≥65-year group as Priority II (management focus), and the <45-year group as Priority III (selective prevention) (**Supplementary Table S8**).

Similar age-dependent mortality risk situations were evident across CKM stages. During median follow-up periods of 7.93 years (Stage 3), 9.00 years (Stage 4), 9.04 years (Stage 4a), and 7.76 years (Stage 4b), 3,876, 3,181, 2,862, and 772 deaths occurred, respectively. The results from studies stratified by CKM revealed trends similar to those in A-CKM overall, with risk estimates for mortality gradually decreasing as the onset age increased. Stage 3 CKM was associated with increased mortality risk across all age groups except for participants aged ≥65 years at disease onset. Notably, Stage 4b CKM showed nonsignificant trends in the youngest cohort, likely because of the limited number of events (**Supplementary Figure S3**).

### 3.3 Stratified analysis of risk factors for all-cause mortality by A-CKM onset age

We evaluated the heterogeneous associations between CKM risk factors and all-cause mortality across age-at-onset subgroups. Subgroup analyses stratified participants by baseline covariates such as smoking status, alcohol consumption, hypertension, diabetes, dyslipidemia, MetS, and systemic inflammation (assessed by hs-CRP and HDL-C levels). Overall, a significant interaction was observed between onset age and mortality risk in all the subgroups (*P* for interaction < 0.001), with mortality risk resembling that of the overall cohort; the highest death risk was observed in the <45-year-onset group, and it decreased progressively with advancing age (**Supplementary Table S9**). Notably, in stage-specific analyses, uneven population distribution limited statistical power, resulting in nonsignificant or unquantifiable associations in certain subgroups (**Supplementary Tables S10–S18**).

#### 3.3.1 Onset age–behavior interactions

Among <45-year-onset A-CKM patients, smokers had a significantly greater risk of all-cause mortality than other age groups did (HR = 5.27, 95% CI 1.54 – 17.98). Nonsmoking young-onset cases showed no excess risk versus controls (*P=*0.094). The mortality risk was not significantly different between A-CKM patients with an onset age <45 years or ≥65 years who consumed alcohol versus controls (*P*>0.05). All the other age-alcohol subgroups demonstrated elevated mortality risk. Notably, among <45-year-onset A-CKM patients, drinkers (HR = 2.71; *P=0.129*) and those who did not smoke or use alcohol (HR = 3.19; P = 0.055) did not have an increased risk, whereas nondrinkers had significantly increased mortality (HR = 4.07; *P* = 0.006). Smoking, regardless of alcohol use, was strongly associated with mortality (HR = 4.17, *P* = 0.010).

#### 3.3.2 Interactions between onset age and comorbidities

Compared with older-onset patients, <45-year-onset A-CKM patients with hypertension (HR = 3.69; *P* = 0.039), dyslipidemia (HR = 4.97; *P* = 0.003), or MetS (HR = 12.89; *P* = 0.013) demonstrated a particularly greater association with mortality. In ≥45-year-onset A-CKM patients, mortality risk remained elevated regardless of comorbidity status. For 45 – 64-year-onset CKM patients, diabetes status was strongly associated with mortality (all HR>1, *P*<0.05). In contrast, diabetes status did not affect the risk in <45-year-onset (HR = 4.96; *P* = 0.151) or ≥65-year-onset (HR = 0.90; *P* = 0.331) A-CKM patients.

#### 3.3.3 Onset age–biomarker interactions

An elevated hs-CRP concentration (≥3 mg/L) was associated with a 10.15-fold increased mortality risk in patients <45-year-onset (95% CI 1.32 – 77.85*; P* = 0.026), whereas those with a hs-CRP concentration <3 mg/L did not have an increased risk (HR = 2.02; *P* = 0.178), whereas a low HDL-C concentration strongly increased mortality (HR = 17.94; *P* = 0.005). Even normal HDL-C levels were significantly more common in the <45 age group (HR = 2.76; *P* = 0.023), whereas in the ≥45 years age group, HDL-C levels were most strongly associated with mortality regardless of HDL-C level (HR>1.13; P<0.001).

#### 3.3.4 Onset age–risk subtype interaction effects

Stage 3 A-CKM patients < 45 years of age with very high-risk CKD had nonsignificant increases in mortality (HR = 2.39; 95% CI: 0.59 – 9.71; *P* = 0.224). However, the high predicted CVD risk in this subgroup resulted in notably higher mortality (HR = 24.06; *P*<0.001), although caution is observed due to a small event number (7 cases). Among ≥45-year-onset Stage 3 patients with CKD/CVD risk, the mortality risk significantly increased, although the risk decreased with advancing age. Notably, in patients with CVD onset ≥65 years, the risk of CVD was not significantly different (HR = 1.01, *P* = 0.836) (**Table 2**).

**Table 2:** Differential mortality in stage 3 CKM: competing risks of CKD-dominant vs. CVD-dominant phenotypes.

Onset age (years)	Control subjects		Case subjects		Hazard ratio (95% CI)	*P* value
	Event/Total	Rate	Event/Total	Rate		
New-onset CKM syndrome Stage 3 with very high-risk CKD *(P* for interaction = 0.009)						
<45	4/1070	0.45	7/1002	0.83	2.39 (0.59 - 9.71)	0.224
45-54	37/951	4.08	46/483	10.63	3.25 (2.06 - 5.13)	<0.001
55-64	263/3421	8.93	98/511	22.32	2.92 (2.28 - 3.74)	<0.001
≥65	1442/5924	30.26	106/288	46.29	1.96 (1.60 - 2.41)	<0.001
New-onset CKM syndrome Stage 3 with high predicted CVD risk *(P* for interaction < 0.001)						
<45	4/1070	0.45	7/68	11.77	24.06 (3.94 - 146.92)	<0.001
45-54	37/951	4.08	57/476	12.70	2.82 (1.80 - 4.41)	<0.001
55-64	263/3421	8.93	370/2954	14.98	1.66 (1.41 - 1.97)	<0.001
≥65	1442/5924	30.26	1507/5723	31.99	1.01 (0.93 - 1.09)	0.836

Data presented as hazard ratio (95% confidence interval), with event rates per 100 person-years.

P values for phenotype-age interaction terms: very high-risk CKD cohort = 0.009, high predicted CVD risk cohort < 0.001. Control subjects: Age-matched individuals without cardio-kidney-metabolic dysfunction. Case subjects: New-onset Stage 3 CKM patients stratified by predominant phenotype.

### 3.4 Population attribution fractions and number needed to treat

Consistent with the results of Cox regression analyses, the population-attributable mortality burden in A-CKM patients decreased significantly with increasing onset age, demonstrating a monotonic decline from 56.14% in those with onset before 45 years to 41.37% at 45 – 54 years, 32.50% at 55 – 64 years, and 8.57% at ≥65 years. This striking 67.7% relative reduction in attributable burden (56.14%→8.57%)—reflecting the highest burden in younger-onset cohorts—was consistently observed across all CKM disease stages (**Supplementary Figure S2**).

Quantification of intervention efficiency revealed striking age-dependent variation (**Table 3**), with the 45 – 54-year onset group (NNT = 15) demonstrating an optimal cost–benefit ratio—treating 15 individuals for 10 years prevented one death—compared with younger (NNT = 56) and older groups (NNT = 18). With respect to quantification of intervention efficiency using NNT, the 55 – 64-year group (NNT = 12) showed comparable efficacy but later clinical impact.

**Table 3:** Age-stratified mortality risk and intervention efficiency in A-CKM.

Onset age	A-CKM mortality rate (/1000 PY)	Control mortality rate (/1000 PY)	Absolute risk difference (/1000 PY)	10-year NNT (95% CI)
<45 years	2.48	0.69	1.79	56 (40 – 85)
45–54 years	11.11	4.36	6.75	15 (12 – 20)
55–64 years	17.24	8.63	8.61	12 (9 – 16)
≥65 years	33.95	28.29	5.66	18 (15 – 22)

Mortality rates: Expressed per 1,000 person-years (PY); Number needed to treat (NNT) calculation: NNT = 1000/(absolute risk difference × 10), representing patients needing 10-year intervention to prevent one death; absolute risk difference (ARD)=95% confidence intervals (CIs): computed via Fieller’s theorem accounting for distribution properties of ratio metrics.

### 3.5 Sensitivity analyses

We performed multiple sensitivity analyses by sequentially excluding (1) participants with mortality events occurring within the first year of follow-up, (2) participants engaging in regular physical activity, and (3) participants with medication histories. Despite these exclusions, the age-stratified mortality trends remained consistent across all A-CKM stages, with findings aligning closely to the main analysis results (**Supplementary Table S19**).

Given the male predominance (89.4%) in our cohort—reflecting higher A-CKM incidence in males—we performed sex-stratified sensitivity analyses (**Supplementary Table S20**). According to sex-stratified analyses, females exhibited substantially increased midlife vulnerability (45 – 54 y onset: Stage 4 HR = 14.25, 95% CI 3.77 – 54.89 vs. male HR = 2.54) and persistently elevated elderly risk (≥65 y: mean HR 48% higher than males). These critical sex-specific patterns emerged despite the limited statistical power in early-onset female subgroups (<45 y), where wider confidence intervals (e.g., HR = 0.95, 95% CI: 0.13 – 7.11) precluded definitive interpretation because of the smaller sample size (n=3,664 females vs. 30,902 males).

## 4 Discussion

In this large prospective cohort, we observed a strong age-dependent association between A-CKM and all-cause mortality: patients diagnosed before age 45 exhibited the highest relative hazard (HR = 3.35), although this attenuated with older onset—particularly beyond age 65 (HR = 1.17). A pivotal paradox emerged in disease burden metrics: young-onset patients (<45 y) demonstrated a low absolute mortality burden (ΔRate +1.49/1000 PY) but the highest PAF (56.14%), indicating maximal population-level prevention potential. Conversely, late-onset patients (≥65 y) had a substantially greater absolute burden (ΔRate +4.55/1000 PY) but minimal PAF (8.57%), necessitating individualized management. Most critically, we identified the 45 – 54-year onset window as having optimal intervention efficiency (NNT = 15 over 10 years), fundamentally shifting preventive focus to this high-yield transition period where systematic screening prevents one death per 15 patients treated.

The steep age-dependent attenuation of A-CKM-associated mortality risk—manifested in the HR decline from 3.35 (onset <45y) to 1.17 (≥65y)—parallels CARDIA’s documentation of exponentially rising coronary pathology in midlife (25). Crucially, our identification of peak intervention efficiency during onset ages 45 – 54 years (NNT = 15/10y) dovetails with CARDIA’s observed coronary calcium surge at ages 40 - 50, establishing this decade as the prime screening window. This gradient fundamentally revises earlier risk paradigms: whereas traditional cardiometabolic models suggested uniformly low absolute risk in young adults (26), our mortality quantification reveals substantial vulnerability in young A-CKM patients through disease-stage specificity mechanisms. Specifically, multiorgan amplification drives mortality elevation beyond single-system disorders, evidenced by the 3.35-fold excess risk in young A-CKM patients versus 1.6-fold in T2DM (17). Further, the acceleration of biological aging processes—mirrored in diabetes cohorts where early diagnosis amplifies cardiovascular decline (27)—explains this differential risk. Critically, cross-disease validation confirms consistent patterns, with hypertension studies demonstrating <45y diagnoses confer 2.59-fold mortality risk (28) and T2DM cohorts showing ≤40y onset carries OR 2.05 mortality risk (29). The observed 22% per-decade risk attenuation aligns with 25 - 40% reductions documented across cardiometabolic diseases per diagnostic delay decade, confirming this attenuation as a fundamental biological phenomenon transcending disease boundaries.

The most compelling evidence for targeted intervention emerges from our integrated risk assessment [HR, ΔRate, NNT (30)], delineating an age-stratified strategy with four distinct management priorities. This paradoxical dissociation—where young-onset patients exhibit high PAF but low ΔRate, while late-onset patients show inverse patterns—aligns with competing risk stratification theory (31). Specifically, minimal background mortality in younger cohorts (<45 y) amplifies A-CKM’s detectable population impact, whereas converging comorbidities in elderly (≥65 y) obscure its isolated contribution (32). Our analysis identifies two critical intervention windows: the 45 – 54-year cohort represents the optimal prevention efficiency window (NNT = 15), where primary interventions yield maximal return per resource invested; concurrently, the 55 – 64-year cohort constitutes the burden reduction window (ΔRate +8.61/1,000 PY), demanding intensive management to mitigate accumulated progression. Although the latter group shows numerically superior NNT (12 vs. 15), this reflects demographic artifacts—elevated background mortality amplifying absolute risk reduction—rather than genuine efficacy gains. Consequently, delaying intervention until this stage forfeits preventable life-years achievable through earlier interception. Resource allocation must thus differentiate these goals: preventive resources (e.g., screening) should prioritize the 45 – 54-year window to forestall irreversible organ damage, while treatment intensification targets the 55 – 64-year cohort to address their elevated mortality burden. This dual-window strategy resolves the efficiency-burden paradox by maximizing life-years gained and mortality reduction across the A-CKM continuum. Crucially, while NNT provides practical efficiency metrics where cost analyses prove infeasible (33), its apparent superiority in 55 – 64-year group confirms—rather than contradicts—the 45 – 54-year window’s primacy as the optimal resource target. These findings consolidate into a precision framework where management priorities evolve across the lifespan: population prevention (<45 y), efficiency-driven interception (45 – 54 y), individualized management (55 – 64 y), and comorbidity optimization (≥65 y).

Multiple studies have characterized age-dependent cardiometabolic risk trajectories, and our quantification of a 22% per-decade mortality risk attenuation extends prior evidence through three key advances. Regarding young-onset (<45y) risk amplification, A-CKM staging demonstrated 186% excess hazard—surpassing established benchmarks such as type 2 diabetes (+130% versus OR 2.05) (29) and hypertension (+126% versus HR 2.59) (20). This divergence underscores how multiorgan pathology in A-CKM potentiates mortality risk beyond single-system disorders. For midlife (45 - 54y) intervention efficacy, our A-CKM staging achieved superior efficacy (NNT = 15 vs. diabetes models’ +2.8% risk difference) (34), enabling 12-year-earlier intervention than conventional paradigms. Concurrently, biological aging mediation analysis revealed that KDM-BA acceleration (OR 1.44) (35) paralleled our attenuation gradient, with its peak mediation effect in the 45 - 65y cohort aligning precisely with our identified efficiency window. Furthermore, A-CKM staging demonstrates validated superiority over emerging metabolic indices. The METS-IR index, while capturing metabolic dysfunction (HR 1.38 for mortality) (36), exhibited substantially lower precision. This advantage originates from direct pathophysiological anchoring: where METS-IR depends on phenotypic-age-mediated mechanisms explaining about 50% of risk, A-CKM directly quantifies renal dysfunction trajectories, metabolic dysregulation via glycemic/lipid biomarkers, and vascular injury patterns. By internalizing biological aging processes that other indices merely correlate with, A-CKM transforms theoretical risk into actionable intervention pathways.

With respect to risk factors, our study establishes A-CKM as a critical syndromic entity strongly associated with renal dysfunction, metabolic syndrome (MetS), and systemic inflammation. CKM predominantly arises from adipose tissue excess or dysfunction, where metabolic obesity—rather than BMI alone—serves as the critical CVD risk determinant (37, 38). The observed age-dependent heterogeneity in mortality risk is driven by complex interactions among behavioral factors, comorbidities, and inflammatory pathways. In early-onset patients (<45 years), we identified a metabolic-inflammatory paradox: despite having the lowest mean BMI (25.30 ± 3.81 kg/m²) and systolic blood pressure (130.18 ± 21.21 mmHg), this cohort exhibited the highest levels of systemic inflammation (median hs-CRP 1.64 mg/L) and detrimental behaviors (current smoking 49.48%; alcohol use 52.47%). This pathophysiology illuminates our key finding: young-onset patients with lowest BMI yet severe inflammation exceed traditional obesity-duration risk models [HR 1.03/year in CARDIA (39)]. TyG-WHtR indices’ superiority over BMI [CVD mortality HR = 1.66 (40)] and CRP-heart failure kinkage [HR 1.10/mg/L (41)] confirming inflammation-metabolism synergy as the primary driver. Congruent with this pathophysiology, our cohort documented that the paradoxical risk signature in young-onset A-CKM patients potentially implicates visceral adiposity-triggered inflammation—as substantiated by TNF-α mediation explaining 62% of metabolic variance in analogous cohorts (42)—thereby warranting strategic consideration of insulin resistance-targeted interventions (TyG-BMI OR 2.37, p<0.001 (42) concurrent with weight management. Their mortality risk is critically amplified by modifiable factors including smoking (HR = 5.27), hs-CRP ≥3 mg/L (HR = 10.15), HDL-C <1.3 mmol/L (HR = 17.94), and comorbidities (hypertension HR = 3.69; dyslipidemia HR = 4.97; MetS HR = 12.89). Importantly, mortality risk approached control levels in the absence of these amplifiers, revealing a pivotal prevention window where aggressive targeting of these factors—particularly smoking cessation, cardiometabolic control, and inflammation suppression (hs-CRP <2 mg/L) (43, 44)—may normalize risk. Alcohol use: Mortality risk followed a U-shaped pattern: neutral in patients <45 and ≥65 years, but elevated at 45 – 64 years, and the paradoxical elevation of risk among nondrinking young A-CKM patients necessitate cautious interpretation (45). Regardless of HDL-C levels, the mortality risk of A-CKM patients significantly increases across all ages at onset, highlighting the complex role of HDL-C, which requires further exploration (46).

This contrasts sharply with the 45 – 54-year transition cohort, where metabolic decompensation manifests through the highest triglyceride levels (median 1.55 mmol/L), rapid hypertension progression (66.19% vs. 29.87% in <45y), diabetes surge (25.05% vs. 8.08%), and antihypertensive medication initiation (24.45%). While A-CKM itself drove substantial mortality risk (HR = 2.58), modifiers including diabetes (HR = 2.29) and hs-CRP ≥3 mg/L (HR = 3.32) remained potent. This transitional period represents a high-yield intervention window for preventing full decompensation. Critically, Asian CKM patients exhibit ectopic fat distribution patterns (47, 48), with visceral adipose accumulation driving proinflammatory factor secretion that synergistically aggravates metabolic dysregulation, vascular impairment, and renal dysfunction at diagnosis (4).

Systemic low-grade inflammation (hs-CRP > 2.0 mg/L) independently predicts CVD (49) and all-cause mortality (50), mediated through endothelial dysfunction and thrombosis (51)—with cardiovascular mortality linkage established as ‘convincing evidence’ in meta-reviews (52). Critically, —starkly exceeding the 1.6-fold risk in diabetic cohorts (44)—establishing its unique predictive potency in youth. Paradoxically elevated HDL-C alongside inflammation in younger patients (53) reflects HDL dysfunction during inflammatory stress (54), necessitating inflammation-first targeting. Age-related inflammatory escalation exacerbates cardiorenal vulnerability (55), and given significantly reduced eGFR in youth (<45y: 78 ± 12 vs 93 ± 11 mL/min)(**Supplementary Table S21**), stratified analyses confirmed persistent excess mortality risk even with preserved renal function (eGFR ≥60 mL/min: HR = 6.33, 2.50 – 16.05; P<0.001). This demonstrates renal impairment and metabolic derangement coexist as independent drivers of adverse outcomes (56) rather than sequential events—supporting the “double-hit hypothesis” (57) whereby early metabolic insults synergize with inflammation to drive high-risk CKM phenotype. For accelerated renal decline in 45 - 49y patients (58), KDIGO 2024 combined eGFR/urine albumin-to-creatinine ratio (UACR) assessment proves critical (59).

Therapeutic priorities diverge sharply by age: Shenzhen cohort data mandate intensive modifiable risk control for <65y (weight loss >10%: mortality HR 1.41 - 1.79) versus stability-focused preservation for ≥65y (weight fluctuation >10%: HR 1.13 - 1.69) (60). PREDICT cohort evidence further specifies youth require lifestyle-driven interception while elderly prioritize functional resilience (61). Intervention efficacy aligns with this dichotomy: preventing A-CKM in youth (<45y) may reduce population mortality burden by 56.14%, while renal protection dominates for ≥65y (62). Subgroup analyses validate younger high-risk patients face catastrophic mortality (HR = 24.06) whereas stage 3 CKD retains prognostic significance in the elderly (HR = 1.96). As conventional CVD risks attenuate with aging (e.g., stage 3 CVD HR = 1.01 in ≥65y), comprehensive management prioritizing renoprotection (eGFR<80 mL/min) (63), lifestyle optimization, and CKD control becomes imperative amid pervasive risk saturation. However, age-specific neutral risk associated with diabetes may be related to competing risks or limited power and requires further study (64).

Sex-stratified analyses validate established biological differences in A-CKM mortality trajectories across the lifespan. Crucially, age at onset remains the dominant organizer of risk management, with critical convergence points. Notably, a premenopausal paradox emerges wherein younger females (<45 years) exhibit attenuated A-CKM mortality risk, potentially reflecting estrogen-mediated cardiorenal protection (65)—however, this does not negate shared risk containment phase priorities for both sexes, notably smoking cessation and systemic inflammation control. The midlife transition (45 – 54 years) constitutes a universally critical prevention efficiency window that demands intensive metabolic intervention; however, females require heightened vigilance against menopausal-associated risk escalation (HR = 14.25 for Stage 4 CKM) (66). In elderly patients (≥65 years), despite sex-mediated differences in vascular senescence rates (67), renal optimization emerges as the supreme objective for all elderly patients. Thus, while biological sex modulates tactical priorities within age strata, the age-stratified management framework remains universally applicable.

Integrated multidimensional evidence—including HR gradients, ΔRate, NNT, PAF, and distinct age-stratified risk factor profiles—supports a precision management matrix fundamentally organized by age stratification and augmented through sex-specific modifications reflecting established physiological divergence. For early-onset cases (<45 y; risk containment phase), universal priorities target modifiable amplifiers: smoking cessation and stringent hypertension control (SBP<130 mmHg) (68). Sex-specific augmentations include assessing estrogenic-mediated cardiorenal protection in women while intensifying hs-CRP suppression (<2 mg/L) (69) in men. During the pivotal midlife prevention window (45 – 54 years), systematic screening via metabolic decompensation protocols becomes imperative, concurrent with heightened cardiometabolic vigilance for women traversing the peri-/postmenopausal transition (HR = 14.25 risk elevation). In late-adulthood onset (55 – 64 years; disease management phase), interventions confront peak absolute burden (ΔRate +8.61/1000 person-years) through dynamic renal function surveillance, complemented by sex-tailored strategies: vascular senescence screening in women and sustained inflammation control in men. Among elderly patients (≥65 years; optimization phase), universally prioritized renoprotective comanagement (70) integrates polypharmacy optimization to counter persistently elevated female mortality risk (HR>1.60).

Critically, our cohort identified suboptimal treatment rates for hypertension, diabetes, and hyperlipidemia across all age groups with new-onset A-CKM—most severe in the critical 45 – 54-year demographic where therapeutic burden escalates yet remains inadequate (71). This care gap underscores systemic deficiencies in early CKM recognition and preventive delivery, directly fueling high-risk progression. Consequently, strengthening comorbidity surveillance while ensuring accessible, evidence-based age-stratified pharmacotherapy and lifestyle interventions emerges as an essential mandate for attenuating A-CKM mortality across the lifespan (72).

### 4.1 Strengths

Prospective community-based cohort (N = 179,328) with extended follow-up; rigorous age-stratified analysis using quantitative metrics (HR attenuation, NNT, PAF); and CKM staging validated via incident case-control matching.

### 4.2 Limitations

1) Sex imbalance: Predominant male composition limits female-specific risk exploration despite supplementary stratified analyses. 2) Potential biases: Selection: ≥3-examination requirement may exclude high-risk populations; Recall: Behavioral variables (smoking/alcohol) susceptible despite validation; Pharmacotherapy: Medication documented by class (antihypertensive/hypoglycemic/lipid-lowering) but lacking dosage/adherence data; Misclassification: CKM staging adapted to operational data constraints (73); 3) Temporal resolution: Baseline cross-sectional grouping precludes trajectory analysis; Biennial examinations may miss inter-assessment transitions; and Onset timing lag: Interval-defined diagnosis may lag biological onset for cases with ≥8-year intervals (3% of cohort); 4) Competing risks: Cause-specific mortality data unavailable–particularly relevant for elderly (≥65y) risk attenuation. 5) Translational caution: Observational NNT/attenuation estimates require prospective validation; medication-specific effects unassessed. Future directions: Pharmacogenomic profiling, cause-specific mortality linkage via national registries, female-predominant cohort studies, and targeted trials (e.g., anti-inflammatories for young high-hs-CRP; intensive metabolic control in 45 - 54y) will refine implementation.

## 5 Conclusions

This prospective cohort study identifies advanced cardiovascular–kidney–metabolic (A-CKM) syndrome as a significant predictor of age-stratified mortality, revealing a precision management framework defined by four clinical imperatives: targeted risk interception before age 45 with sex-specific modifications, prioritized screening during the midlife window (45 – 54 years), complication-focused protocols for peak burden years (55 – 64), and comorbidity-calibrated renoprotection in elderly individuals (≥65). These findings suggest that strategic resource allocation toward prevention at 45 – 54 years and therapeutic intensification at 55 – 64 years may address the observed efficiency-burden paradox. Implementation would require developing sex-specific biomarker thresholds, integrating these into tiered care pathways, and validation through management trials—potentially advancing A-CKM care beyond chronological age toward biology-informed precision prevention.

## Data Availability

The original contributions presented in the study are included in the article/**Supplementary Material**. Further inquiries can be directed to the corresponding authors.
