# Supplementary material for: Association between onset age and mortality gradients in advanced cardiovascular–kidney–metabolic syndrome
Source: Front Endocrinol (Lausanne). 2025 Sep 3;16:1648083. doi: 10.3389/fendo.2025.1648083 (PMC12440721; doi:10.3389/fendo.2025.1648083)
Supplement: Supplementary file 1 [file DataSheet1.docx]

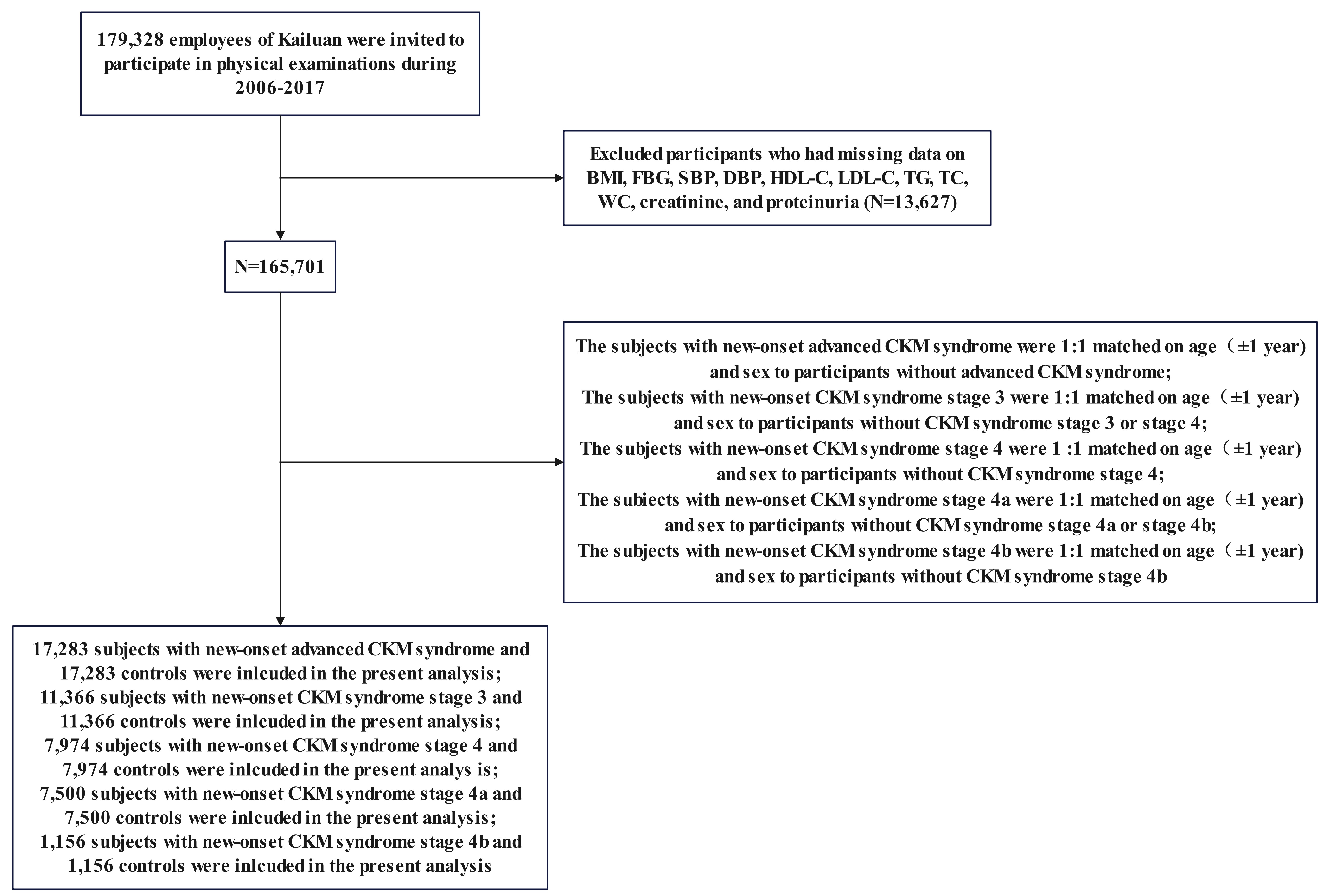


**Fig. S1. Flowchart of the study population.**

Note: CKM, cardiovascular-kidney-metabolic; BMI, body mass index; DBP, diastolic blood pressure; FBG, fasting blood glucose; HDL-C, high-density lipoprotein cholesterol; LDL-C, low-density lipoprotein cholesterol; SBP, systolic blood pressure; TC, total cholesterol; TG, triglycerides; WC, waist circumference.


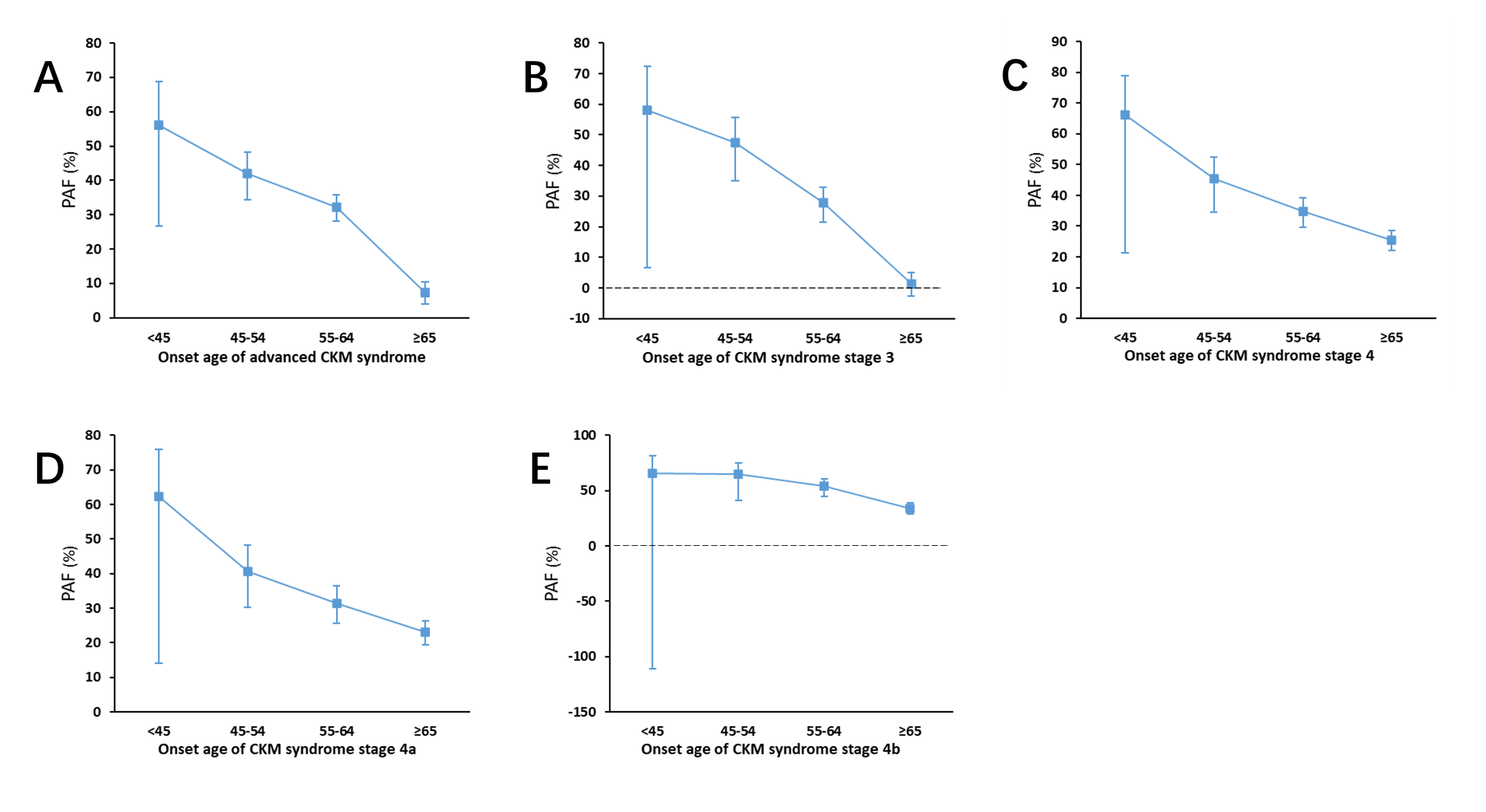


**Fig. S2. Population-attributable fraction of new-onset cases across age-groups for all-cause mortality: Age-dependent attenuation of mortality PAF in A-CKM syndrome, peaking in young-onset cohorts**

Note: Model was adjusted for smoking status, drinking status, physical activity, education, work type, and high-sensitivity C-reactive protein.

CKM, cardiovascular-kidney-metabolic; PAF, Population-attributable fraction.

**Fig. S3. Hazard ratios (95% CI) for all-cause mortality among participants with new-onset advanced CKM stages versus control subjects across age groups**

Note: The rate is per 1,000 person-years. The model was adjusted for smoking status, drinking status, physical activity, education, work type, and high-sensitivity C-reactive protein.

P for interaction, the interaction between cases and their age at diagnosis.

**Table S1. Determination of new-onset** **advanced CKM syndrome**

| 2006-07 | 2008-09 | 2010-11 | 2012-13 | 2014-15 | 2006-07 | 2008-09 | 2010-11 | 2012-13 | 2014-15 | 2016-17 |
| --- | --- | --- | --- | --- | --- | --- | --- | --- | --- | --- |
| 0 | 1 |  |  |  | NA | NA | NA | NA | 0 | 1 |
| NA | 0 | 1 |  |  | NA | NA | NA | 0 | NA | 1 |
| 0 | NA | 1 |  |  | NA | NA | NA | 0 | 0 | 1 |
| 0 | 0 | 1 |  |  | NA | NA | 0 | NA | NA | 1 |
| NA | NA | 0 | 1 |  | NA | NA | 0 | NA | 0 | 1 |
| NA | 0 | NA | 1 |  | NA | NA | 0 | 0 | NA | 1 |
| NA | 0 | 0 | 1 |  | NA | NA | 0 | 0 | 0 | 1 |
| 0 | NA | NA | 1 |  | NA | 0 | NA | NA | NA | 1 |
| 0 | NA | 0 | 1 |  | NA | 0 | NA | NA | 0 | 1 |
| 0 | 0 | NA | 1 |  | NA | 0 | NA | 0 | NA | 1 |
| 0 | 0 | 0 | 1 |  | NA | 0 | NA | 0 | 0 | 1 |
| 0 | 0 | 0 | 0 | 1 | NA | 0 | 0 | NA | NA | 1 |
| 0 | 0 | 0 | NA | 1 | NA | 0 | 0 | NA | 0 | 1 |
| 0 | 0 | NA | 0 | 1 | NA | 0 | 0 | 0 | NA | 1 |
| 0 | 0 | NA | 0 | 1 | NA | 0 | 0 | 0 | 0 | 1 |
| 0 | NA | 0 | 0 | 1 | 0 | NA | NA | NA | NA | 1 |
| 0 | NA | 0 | NA | 1 | 0 | NA | NA | NA | 0 | 1 |
| 0 | NA | NA | 0 | 1 | 0 | NA | NA | 0 | NA | 1 |
| 0 | NA | NA | NA | 1 | 0 | NA | NA | 0 | 0 | 1 |
| NA | 0 | 0 | 0 | 1 | 0 | NA | 0 | NA | NA | 1 |
| NA | 0 | 0 | NA | 1 | 0 | NA | 0 | NA | 0 | 1 |
| NA | 0 | NA | 0 | 1 | 0 | NA | 0 | 0 | NA | 1 |
| NA | 0 | NA | NA | 1 | 0 | 0 | 0 | 0 | 0 | 1 |
| NA | NA | 0 | 0 | 1 | 0 | 0 | NA | NA | NA | 1 |
| NA | NA | NA | 0 | 1 | 0 | 0 | NA | NA | 0 | 1 |
| NA | NA | 0 | NA | 1 | 0 | 0 | NA | 0 | NA | 1 |
|  |  |  |  |  | 0 | 0 | NA | 0 | 0 | 1 |
|  |  |  |  |  | 0 | 0 | 0 | NA | NA | 1 |
|  |  |  |  |  | 0 | 0 | 0 | NA | 0 | 1 |
|  |  |  |  |  | 0 | 0 | 0 | 0 | NA | 1 |
|  |  |  |  |  | 0 | 0 | 0 | 0 | 0 | 1 |

Note:

NA: The participants did not come to the hospital for medical examination in this cycle.

0: The participants participated in this round of physical examination, but was not diagnosed as advanced CKM syndrome.

1: The participants participated in this round of physical examination and was diagnosed as advanced CKM syndrome.

Appendix Note 1: Traditional Matching Specifications

Case-control matching was performed with the following criteria:

- Age matching: ±1-year range

- Disease status: Exact absence of A-CKM in all prior examinations

- Temporal matching: Exact calendar-year enrollment

- Sex matching: Exact match

Appendix Note 2: Matching Tolerance Rationale

The matching parameters (±1-year age, exact calendar year) were implemented based on:

- Biological plausibility: Cardiometabolic risk profiles show high comparability within ±1-year age cohorts

- Operational optimization: Balancing match pair yield with temporal homogeneity, optimized for biennial examination intervals

**Table S2. Comprehensive Methodological Details of Laboratory Analyses**

| Biomarker | Method (Principle) | Manufacturer (Catalog) | Range | CV (%) | Calibration Standard |
| --- | --- | --- | --- | --- | --- |
| FBG | Hexokinase/G6PDH enzymatic | MindBio (GA-0031) | 0.5-30.07 mmol/L | Intra:1.2 Inter:2.5 | NIST SRM 965b |
| HDL-C | PEG-modified enzyme colorimetry | Roche (06657511) | 0.08-3.10 mmol/L | Intra:<3 Inter:<5 | CDC RM 026 |
| LDL-C | Selective detergent protection | Roche (07027026) | 0.10-10.35 mmol/L | Intra:<2.5 Inter:<4 | CDC RM 026 |
| TC | Cholesterol oxidase-H₂O₂ endpoint | Sekisui (292-65804) | 0.50-20.70 mmol/L | Intra:<2 Inter:<3 | CRMLN 2021A |
| TG | GPO-Trinder method | Wako (461-08992) | 0.10-11.30 mmol/L | Intra:<3 Inter:<5 | CDC RM 027 |
| Creatinine | Sarcosine oxidase-PAP | BioSino (CD1002) | 8.8-1768 μmol/L | Intra:<2 Inter:<3 | ERM-DA252k (IDMS) |
| hs-CRP | Latex-enhanced immunoturbidimetry | Kanto (CPH66) | 0.1-20 mg/L | Intra:<5 Inter:<8 | WHO 85/506 |

Note: The coefficients of variation (CV) reflect empirical measurements obtained during the study: intra-assay CV quantifies precision within individual analytical runs performed daily, while inter-assay CV represents precision consistency across consecutive runs, with the "<" symbol denoting maximum observed variation values. Methodological performance verification confirmed the LDL-C assay maintained analytical precision across the triglyceride detection range of 0.10-11.30 mmol/L. Gender-specific diagnostic thresholds were implemented per protocol, notably applying the female HDL-C cut-off of <1.29 mmol/L consistent with metabolic syndrome diagnostic criteria. All biomarker analyses were completed within the 4-hour post-collection window as operationally executed throughout the investigation. Extended methodological details for urinalysis parameters, including DIRUI N-600 analyzer specifications and Bio-Rad quality control materials.

Rigorous quality management protocols were implemented throughout the study. During the pre-analytical phase, ambient conditions were maintained at 22±2°C with 40-60% relative humidity using medical-grade HVAC systems to ensure sample stability. The analytical phase included daily internal quality control procedures with Bio-Rad control materials (urinalysis), complemented by biweekly instrument calibration adhering to ISO 15189:2012 standards. Post-analytical verification was achieved through participation in external quality assessment programs administered by the National Center for Clinical Laboratories, with all parameters demonstrating >90% proficiency testing scores.

**Table S3. Definition of CKM syndrome stages.**

| CKM syndrome stages | Definition (AHA Original Criteria) | Current Study Adaptation | Rationale |
| --- | --- | --- | --- |
| Stage 0: No CKM risk factors | Individuals with normal BMI and waist circumference, normoglycemia, normotension, a normal lipid profile, and no evidence of CKD or subclinical or clinical CVD |  |  |
| Stage 1: Excess or dysfunctional adiposity | Individuals with overweight/obesity, abdominal obesity, or dysfunctional adipose tissue, without the presence of other metabolic risk factors or CKD  BMI ≥25 kg/m^2^ (or ≥23 kg/m^2^ if Asian ancestry), Waist circumference ≥88/102 cm in women/ men (or if Asian ancestry ≥80/90 cm in women/ men), or Fasting blood glucose ≥100–124 mg/dL or HbA1c between 5.7% and 6.4% | Excluded HbA1c between 5.7% and 6.4% | Operational constraint: HbA1c unavailable in 98.7% cohort → Alternative validation: Fasting glucose ≥100 mg/dL predicted incident diabetes |
| Stage 2: Metabolic risk factors and CKD | Individuals with metabolic risk factors (hypertriglyceridemia [≥135 mg/dL], hypertension, MetS, diabetes), or CKD |  |  |
| Stage 3: Subclinical CVD in CKM | Subclinical ASCVD or subclinical HF among individuals with excess/dysfunctional adiposity, other metabolic risk factors, or CKD  •Subclinical ASCVD to be principally diagnosed by coronary artery calcification (subclinical atherosclerosis by coronary catheterization/CT angiography also meets criteria)  •Subclinical HF diagnosed by elevated cardiac biomarkers (NT-proBNP ≥125 pg/mL, hs-troponin T ≥14 ng/L for women and ≥22 ng/L for men, hstroponin I ≥10 ng/L for women and ≥12 ng/L for men) or by echocardiographic parameters, with a combination of the 2 indicating highest HF risk.  Risk equivalents of subclinical CVD  •Very high-risk CKD (stage G4 or G5 CKD or very high risk per KDIGO classification)  •High predicted 10-y CVD risk | 1.For CKM Stage 3, given data limitations, we used risk equivalents of subclinical CVD to define this stage, with CVD risk calculated using the China-PAR model (Prediction for Atherosclerotic Cardiovascular Disease Risk in China);  2.BMI/Waist cutoffs: Universal thresholds→Asian-specific thresholds  3.Not applied NT-proBNP thresholds | 1. China-PAR 10-year CVD risk is a risk prediction tool specific to the Chinese population. 2. AHA recommendation for Asian cohorts 3. Biomarker unavailable |
| Stage 4: Clinical CVD in CKM | Clinical CVD (coronary heart disease, HF, stroke, peripheral artery disease, atrial fibrillation) among individuals with excess/dysfunctional adiposity, other CKM risk factors, or CKD  Stage 4a: no kidney failure  Stage 4b: kidney failure present |  |  |

Note: In this study, the definition of CKM is based on the American Heart Association’s criteria, but there are several key differences to note:

• The cut-off values for BMI and waist circumference follow the AHA-defined Asian criteria.

• ASCVD indicates atherosclerotic cardiovascular disease; BMI, body mass index; CKD, chronic kidney disease; CKM, cardiovascular-kidney-metabolic; CT, computed tomography; CVD, cardiovascular disease; HbA1c, hemoglobin A1c; HDL, high-density lipoprotein; HF, heart failure; hs-troponin, high-sensitivity troponin; KDIGO, Kidney Disease Improving Global Outcomes; MetS, metabolic syndrome; and NT-proBNP; N-terminal pro-B-type natriuretic peptide.

Individuals with gestational diabetes should receive intensified screening for impaired glucose tolerance after pregnancy.

MetS is defined by the presence of 3 or more of the following: (1) waist circumference ≥88 cm for women and ≥102 cm for men (≥80 cm for women and ≥90 cm for men if Asian ancestry); (2) HDL cholesterol <40 mg/dL for men and <50 mg/dL for women; (3) triglycerides ≥150 mg/dL; (4) elevated blood pressure (systolic blood pressure ≥130 mmHg or diastolic blood pressure ≥80 mmHg and/or use of antihypertensive medications); and (5) fasting blood glucose ≥100 mg/dL.

**Table S4. Basic characteristics for participants with new-onset** **CKM syndrome stage 3 and their controls.**

| **Characteristics** | **Control subjects** | **New-onset CKM syndrome stage 3** | ***P* value** | **CKM syndrome stage 3 onset age (years) ^a^** | | | | ***P* for trend** |
| --- | --- | --- | --- | --- | --- | --- | --- | --- |
|  |  |  |  | **<45** | **45-54** | **55-64** | **≥65** |  |
| No. of participants | 11366 | 11366 | / | 1070 | 951 | 3421 | 5924 | / |
| Age, years | 62.44 ± 13.67 | 62.44 ± 13.67 | - | 29.17 ± 14.01 | 50.85 ± 2.89 | 60.93 ± 2.68 | 71.29 ± 4.36 | - |
| Men, n (%) | 10603 (93.29) | 10603 (93.29) | - | 952 (88.97) | 845 (88.85) | 3249 (94.97) | 5557 (93.80) | <0.001 |
| Current drinkers, n (%) | 3055 (30.95) | 3348 (30.59) | 0.575 | 583 (54.95) | 483 (51.06) | 1092 (33.04) | 1190 (21.12) | <0.001 |
| Current smokers, n (%) | 3541 (31.15) | 3472 (30.55) | 0.322 | 529 (49.44) | 525 (55.21) | 1331 (38.91) | 1087 (18.35) | <0.001 |
| Physical activity, n (%) | 1910 (18.51) | 1988 (17.88) | 0.233 | 76 (7.11) | 110 (11.62) | 620 (18.54) | 1182 (20.52) | <0.001 |
| High school or above, n (%) | 1693 (14.90) | 1585 (13.95) | 0.041 | 489 (45.70) | 216 (22.71) | 320 (9.35) | 560 (9.45) | <0.001 |
| Mental work, n (%) | 1633 (14.99) | 1610 (14.26) | 0.167 | 198 (18.52) | 124 (13.07) | 371 (10.94) | 917 (15.58) | <0.001 |
| WC, cm | 87.42 ± 9.46 | 88.47 ± 10.55 | <0.001 | 86.60 ± 10.01 | 89.17 ± 10.76 | 89.12 ± 10.34 | 88.31 ± 10.68 | <0.001 |
| BMI, kg/m^2^ | 24.53 ± 3.26 | 25.59 ± 3.33 | <0.001 | 25.06 ± 3.67 | 25.92 ± 3.43 | 25.95 ± 3.17 | 25.42 ± 3.31 | <0.001 |
| SBP, mmHg | 134.82 ± 17.99 | 158.95 ± 23.46 | <0.001 | 126.97 ± 16.69 | 143.20 ± 27.70 | 152.34 ± 25.21 | 147.73 ± 22.02 | <0.001 |
| DBP, mmHg | 82.03 ± 10.37 | 89.89 ± 13.14 | <0.001 | 80.56 ± 11.03 | 89.08 ± 14.15 | 89.48 ± 12.76 | 84.39 ± 11.57 | <0.001 |
| FBG, mmol/L | 5.65 ± 1.86 | 6.87 ± 2.94 | <0.001 | 5.52 ± 2.79 | 6.76 ± 2.57 | 7.63 ± 3.41 | 6.70 ± 2.59 | <0.001 |
| LDL-C, mmol/L | 2.73 ± 1.29 | 3.02 ± 1.22 | <0.001 | 3.06 ± 1.56 | 3.08 ± 2.05 | 3.10 ± 1.16 | 2.95 ± 0.98 | <0.001 |
| HDL-C, mmol/L | 1.48 ± 0.66 | 1.32 ± 0.54 | <0.001 | 1.54 ± 1.13 | 1.37 ± 0.59 | 1.26 ± 0.41 | 1.30 ± 0.40 | <0.001 |
| TG, mmol/L | 1.12 (0.80, 1.67) | 1.46 (1.02, 2.25) | <0.001 | 1.42 (0.99, 2.34) | 1.84 (1.18, 3.45) | 1.69 (1.18, 2.61) | 1.33 (0.97, 1.96) | <0.001 |
| TC, mmol/L | 4.92 ± 1.16 | 5.35 ± 1.31 | <0.001 | 4.99 ± 1.12 | 5.28 ± 1.41 | 5.30 ± 1.34 | 5.04 ± 1.19 | <0.001 |
| hs-CRP, mg/L | 1.06 (0.44, 2.59) | 1.50 (0.62, 3.10) | <0.001 | 1.70 (1.20, 2.50) | 1.70 (0.81, 3.20) | 1.34 (0.60, 2.94) | 1.43 (0.56, 3.40) | <0.001 |
| eGFR, mL/min/1.73m^2^ | 88.10 ± 20.47 | 72.09 ± 29.91 | <0.001 | 24.98 ± 21.59 | 59.32 ± 38.59 | 80.43 ± 26.90 | 77.23 ± 17.82 | <0.001 |
| Family history of CVD, n (%) | 229 (2.21) | 175 (1.54) | <0.001 | 26 (2.45) | 45 (4.73) | 66 (1.93) | 38 (0.64) | <0.001 |
| MetS, n (%) | 3466 (30.49) | 6322 (55.62) | <0.001 | 314 (29.35) | 585 (61.51) | 2301 (67.26) | 3122 (52.70) | <0.001 |
| Hypertension, n (%) | 5198 (45.73) | 9362 (82.37) | <0.001 | 270 (25.23) | 692 (72.77) | 3176 (92.84) | 5224 (88.18) | <0.001 |
| Diabetes, n (%) | 854 (7.51) | 4247 (37.37) | <0.001 | 68 (6.36) | 326 (34.28) | 1795 (52.47) | 2058 (34.74) | <0.001 |
| Dyslipidemia, n (%) | 6481 (57.02) | 8422 (74.10) | <0.001 | 632 (59.07) | 758 (79.71) | 2813 (82.23) | 4219 (71.22) | <0.001 |
| Anti-hypertension drugs, n (%) | 589 (5.18) | 1439 (12.66) | <0.001 | 17 (1.59) | 149 (15.67) | 484 (14.15) | 789 (13.32) | <0.001 |
| Hypoglycemic drugs, n (%) | 230 (2.11) | 995 (8.75) | <0.001 | 4 (0.37) | 71 (7.47) | 401 (11.72) | 519 (8.76) | <0.001 |
| Lipid-lowering drugs, n (%) | 34 (0.31) | 73 (0.64) | <0.001 | 3 (0.28) | 10 (1.05) | 21 (0.61) | 39 (0.66) | 0.190 |

Note: Data are presented as mean ± SD, median (interquartile range), or n (%).

BMI, body mass index; CVD, cardiovascular disease; CKD, chronic kidney disease; DBP, diastolic blood pressure; eGFR, estimated glomerular filtration rate; FBG, fasting blood glucose; HDL-C, high-density lipoprotein cholesterol; hs-CRP, high-sensitivity C reactive protein; LDL-C, low-density lipoprotein cholesterol; MetS, metabolic syndrome; SBP, systolic blood pressure; SD, standard deviation; TC, total cholesterol; TG, triglycerides; WC, waist circumference.

^a^Basic characteristics for new-onset CKM syndrome stage 3 participants across age groups.

**Table S5. Basic characteristics for participants with new-onset CKM syndrome stage 4 and their controls.**

| **Characteristics** | **Control subjects** | **New-onset CKM syndrome stage 4** | ***P* value** | **CKM syndrome stage 4 onset age (years)^a^** | | | | ***P* for trend** |
| --- | --- | --- | --- | --- | --- | --- | --- | --- |
|  |  |  |  | **<45** | **45-54** | **55-64** | **≥65** |  |
| No. of participants | 7974 | 7974 | / | 275 | 1437 | 3126 | 3136 | / |
| Age, years | 62.56 ± 9.95 | 62.56 ± 9.95 | - | 39.57 ± 5.21 | 51.16 ± 2.77 | 60.11 ± 2.79 | 71.28 ± 5.65 | - |
| Men, n (%) | 6848 (85.89) | 6848 (85.89) | - | 246 (89.45) | 1238 (86.15) | 2651 (84.80) | 2733 (87.15) | 0.022 |
| Current drinkers, n (%) | 2221 (27.85) | 2038 (25.56) | 0.130 | 123 (47.13) | 533 (37.67) | 835 (27.75) | 547 (18.49) | <0.001 |
| Current smokers, n (%) | 2293 (28.76) | 2275 (28.53) | 0.585 | 137 (49.82) | 605 (42.07) | 914 (29.15) | 619 (19.66) | <0.001 |
| Physical activity, n (%) | 1583 (20.31) | 1954 (24.97) | <0.001 | 49 (17.88) | 254 (17.76) | 802 (26.16) | 849 (27.78) | <0.001 |
| High school or above, n (%) | 1125 (14.11) | 1122 (14.07) | 0.883 | 147 (53.45) | 311 (21.63) | 326 (10.40) | 338 (10.73) | <0.001 |
| Mental work, n (%) | 1108 (14.18) | 1151 (14.44) | 0.406 | 49 (17.88) | 172 (11.99) | 373 (11.94) | 557 (17.75) | <0.001 |
| WC, cm | 87.84 ± 9.30 | 89.89 ± 9.91 | <0.001 | 90.38 ± 11.31 | 90.03 ± 9.65 | 90.11 ± 9.73 | 89.58 ± 10.06 | 0.131 |
| BMI, kg/m^2^ | 24.96 ± 3.26 | 25.58 ± 3.41 | <0.001 | 26.22 ± 4.16 | 25.85 ± 3.41 | 25.74 ± 3.31 | 25.24 ± 3.41 | <0.001 |
| SBP, mmHg | 138.41 ± 19.94 | 141.57 ± 20.13 | <0.001 | 127.58 ± 17.43 | 133.88 ± 18.62 | 139.60 ± 19.57 | 144.31 ± 20.26 | <0.001 |
| DBP, mmHg | 84.28 ± 11.11 | 86.01 ± 11.98 | <0.001 | 83.60 ± 11.95 | 86.69 ± 11.85 | 86.13 ± 11.42 | 84.59 ± 11.39 | <0.001 |
| FBG, mmol/L | 5.97 ± 2.29 | 6.24 ± 2.49 | <0.001 | 6.00 ± 3.59 | 6.17 ± 2.78 | 6.31 ± 2.38 | 6.21 ± 2.33 | 0.076 |
| LDL-C, mmol/L | 2.77 ± 1.02 | 2.67 ± 1.44 | <0.001 | 2.51 ± 0.89 | 2.62 ± 0.96 | 2.67 ± 0.93 | 2.71 ± 1.98 | 0.050 |
| HDL-C, mmol/L | 1.47 ± 0.64 | 1.37 ± 0.66 | <0.001 | 1.34 ± 0.41 | 1.39 ± 0.49 | 1.35 ± 0.49 | 1.39 ± 0.82 | 0.0637 |
| TG, mmol/L | 1.25 (0.89, 1.92) | 1.37 (0.97, 2.08) | <0.001 | 1.50 (0.99, 2.45) | 1.45 (1.02, 2.25) | 1.41 (1.00, 2.18) | 1.30 (0.92, 1.90) | <0.001 |
| TC, mmol/L | 5.00 ± 1.66 | 4.84 ± 1.69 | <0.001 | 4.92 ± 2.17 | 4.98 ± 1.60 | 4.85 ± 1.84 | 4.76 ± 1.52 | <0.001 |
| hs-CRP, mg/L | 1.20 (0.50, 2.80) | 1.40 (0.57, 3.30) | <0.001 | 1.30 (0.49, 2.80) | 1.43 (0.60, 3.10) | 1.30 (0.54, 3.10) | 1.50 (0.58, 3.80) | <0.001 |
| eGFR, mL/min/1.73m^2^ | 85.67 ± 19.34 | 84.65 ± 19.66 | 0.001 | 101.58 ± 19.36 | 93.86 ± 20.09 | 86.61 ± 17.42 | 77.01 ± 18.36 | <0.001 |
| Family history of CVD, n (%) | 166 (2.15) | 360 (4.52) | <0.001 | 24 (9.09) | 114 (7.96) | 145 (4.64) | 77 (2.45) | <0.001 |
| MetS, n (%) | 3162 (39.77) | 4077 (50.98) | <0.001 | 139 (50.55) | 732 (50.90) | 1679 (53.56) | 1527 (48.49) | 0.001 |
| Hypertension, n (%) | 4401 (55.35) | 5518 (69.00) | <0.001 | 133 (48.36) | 902 (62.73) | 2162 (68.96) | 2321 (73.71) | <0.001 |
| Diabetes, n (%) | 1224 (15.39) | 1852 (23.16) | <0.001 | 44 (16.00) | 283 (19.68) | 761 (24.27) | 764 (24.26) | <0.001 |
| Dyslipidemia, n (%) | 4975 (62.57) | 5443 (68.06) | <0.001 | 203 (73.82) | 1020 (70.93) | 2182 (69.60) | 2038 (64.72) | <0.001 |
| Anti-hypertension drugs, n (%) | 735 (9.23) | 2310 (28.90) | <0.001 | 67 (24.36) | 448 (31.15) | 884 (28.20) | 911 (28.93) | 0.070 |
| Hypoglycemic drugs, n (%) | 355 (4.50) | 865 (10.82) | <0.001 | 19 (6.91) | 131 (9.11) | 355 (11.32) | 360 (11.43) | 0.013 |
| Lipid-lowering drugs, n (%) | 38 (0.48) | 235 (2.94) | <0.001 | 14 (5.09) | 66 (4.59) | 96 (3.06) | 59 (1.67) | <0.001 |

Note: Data are presented as mean ± SD, median (interquartile range), or n (%).

BMI, body mass index; CVD, cardiovascular disease; CKD, chronic kidney disease; DBP, diastolic blood pressure; eGFR, estimated glomerular filtration rate; FBG, fasting blood glucose; HDL-C, high-density lipoprotein cholesterol; hs-CRP, high-sensitivity C reactive protein; LDL-C, low-density lipoprotein cholesterol; MetS, metabolic syndrome; SBP, systolic blood pressure; SD, standard deviation; TC, total cholesterol; TG, triglycerides; WC, waist circumference.

^a^Basic characteristics for new-onset CKM syndrome stage 4 participants across age groups.

**Table S6. Basic characteristics for participants with new-onset CKM syndrome stage 4a and their controls.**

| **Characteristics** | **Control subjects** | **New-onset CKM syndrome stage 4a** | ***P* value** | **CKM syndrome stage 4a onset age (years)^a^** | | | | ***P* for trend** |
| --- | --- | --- | --- | --- | --- | --- | --- | --- |
|  |  |  |  | **<45** | **45-54** | **55-64** | **≥65** |  |
| No. of participants | 7500 | 7500 | / | 261 | 1370 | 2972 | 2897 | / |
| Age, years | 62.45 ± 9.92 | 62.45 ± 9.92 | - | 39.67 ± 5.10 | 51.16 ± 2.77 | 60.10 ± 2.78 | 72.25 ± 5.66 | - |
| Men, n (%) | 6455 (86.07) | 6455 (86.07) | - | 232 (88.89) | 1180 (86.13) | 2516 (84.66) | 2527 (87.23) | 0.019 |
| Current drinkers, n (%) | 2119 (29.74) | 1939 (27.05) | <0.001 | 119 (48.18) | 508 (37.71) | 805 (28.22) | 507 (18.63) | <0.001 |
| Current smokers, n (%) | 2228 (29.71) | 2156 (28.75) | 0.196 | 130 (49.81) | 576 (42.04) | 881 (29.64) | 569 (19.64) | <0.001 |
| Physical activity, n (%) | 1482 (20.09) | 1856 (25.28) | <0.001 | 47 (18.08) | 244 (17.91) | 763 (26.25) | 802 (28.52) | <0.001 |
| High school or above, n (%) | 1055 (14.07) | 1072 (14.29) | 0.691 | 144 (55.17) | 297 (21.68) | 315 (10.60) | 316 (10.91) | <0.001 |
| Mental work, n (%) | 999 (13.51) | 1066 (14.26) | 0.171 | 46 (17.69) | 162 (11.85) | 348 (11.75) | 510 (17.67) | <0.001 |
| WC, cm | 87.81 ± 9.44 | 89.83 ± 9.87 | <0.001 | 90.17 ± 11.25 | 89.97 ± 9.64 | 90.04 ± 9.70 | 89.51 ± 10.01 | 0.172 |
| BMI, kg/m^2^ | 24.94 ± 3.24 | 25.53 ± 3.37 | <0.001 | 26.12 ± 4.10 | 25.81 ± 3.36 | 25.71 ± 3.30 | 25.17 ± 3.35 | <0.001 |
| SBP, mmHg | 137.99 ± 19.71 | 141.07 ± 19.95 | <0.001 | 126.98 ± 17.05 | 133.99 ± 18.44 | 138.33 ± 19.45 | 143.91 ± 20.02 | <0.001 |
| DBP, mmHg | 84.13 ± 11.21 | 85.81 ± 11.88 | <0.001 | 83.33 ± 12.17 | 86.73 ± 11.82 | 85.89 ± 11.41 | 83.34 ± 11.35 | <0.001 |
| FBG, mmol/L | 5.90 ± 1.81 | 6.14 ± 2.10 | <0.001 | 5.58 ± 1.77 | 5.94 ± 1.96 | 6.08 ± 2.03 | 6.04 ± 1.90 | <0.001 |
| LDL-C, mmol/L | 2.77 ± 0.88 | 2.64 ± 0.91 | <0.001 | 2.52 ± 0.84 | 2.66 ± 0.97 | 2.74 ± 0.90 | 2.71 ± 0.87 | <0.001 |
| HDL-C, mmol/L | 1.46 ± 0.47 | 1.37 ± 0.46 | <0.001 | 1.38 ± 0.43 | 1.43 ± 0.48 | 1.40 ± 0.48 | 1.42 ± 0.46 | 0.002 |
| TG, mmol/L | 1.25 (0.89, 1.91) | 1.36 (0.96, 2.06) | <0.001 | 1.43 (0.94, 2.37) | 1.40 (0.97, 2.16) | 1.35 (0.96, 2.06) | 1.22 (0.88, 1.79) | <0.001 |
| TC, mmol/L | 4.97 ± 1.26 | 4.80 ± 1.34 | <0.001 | 4.81 ± 1.17 | 4.99 ± 1.19 | 4.91 ± 1.33 | 4.82 ± 1.34 | <0.001 |
| hs-CRP, mg/L | 1.18 (0.50, 2.80) | 1.50 (0.62, 3.10) | <0.001 | 1.10 (0.50, 2.62) | 1.26 (0.59, 2.80) | 1.20 (0.50, 2.87) | 1.32 (0.50, 3.30) | <0.001 |
| eGFR, mL/min/1.73m^2^ | 85.72 ± 19.16 | 86.14 ± 18.24 | 0.161 | 102.38 ± 17.80 | 94.99 ± 18.78 | 87.88 ± 15.82 | 78.71 ± 17.04 | <0.001 |
| Family history of CVD, n (%) | 156 (2.14) | 348 (4.66) | <0.001 | 23 (9.20) | 114 (8.36) | 141 (4.76) | 70 (2.42) | <0.001 |
| MetS, n (%) | 2893 (38.57) | 3766 (50.21) | <0.001 | 130 (49.81) | 690 (50.36) | 1578 (53.10) | 1368 (47.22) | <0.001 |
| Hypertension, n (%) | 4094 (54.59) | 5111 (68.15) | <0.001 | 120 (45.98) | 855 (62.41) | 2026 (68.17) | 2110 (72.83) | <0.001 |
| Diabetes, n (%) | 1093 (14.57) | 1643 (21.91) | <0.001 | 38 (14.56) | 259 (18.91) | 684 (23.01) | 662 (22.85) | <0.001 |
| Dyslipidemia, n (%) | 4684 (62.45) | 5081 (67.75) | <0.001 | 190 (72.80) | 967 (70.58) | 2065 (69.48) | 1859 (64.17) | <0.001 |
| Anti-hypertension drugs, n (%) | 687 (9.16) | 2118 (28.24) | <0.001 | 60 (22.99) | 425 (31.02) | 822 (27.66) | 811 (27.99) | 0.025 |
| Hypoglycemic drugs, n (%) | 343 (4.60) | 767 (10.23) | <0.001 | 16 (6.13) | 121 (8.83) | 316 (10.63) | 314 (10.84) | 0.025 |
| Lipid-lowering drugs, n (%) | 37 (0.50) | 225 (3.00) | <0.001 | 13 (4.98) | 66 (4.82) | 93 (3.13) | 53 (1.83) | 0.190 |

Note: Data are presented as mean ± SD, median (interquartile range), or n (%).

BMI, body mass index; CVD, cardiovascular disease; CKD, chronic kidney disease; DBP, diastolic blood pressure; eGFR, estimated glomerular filtration rate; FBG, fasting blood glucose; HDL-C, high-density lipoprotein cholesterol; hs-CRP, high-sensitivity C reactive protein; LDL-C, low-density lipoprotein cholesterol; MetS, metabolic syndrome; SBP, systolic blood pressure; SD, standard deviation; TC, total cholesterol; TG, triglycerides; WC, waist circumference.

^a^Basic characteristics for new-onset CKM syndrome stage 4a participants across age groups.

**Table S7. Basic characteristics for participants with new-onset CKM syndrome stage 4b and their controls.**

| **Characteristics** | **Control subjects** | **New-onset CKM syndrome stage 4b** | ***P* value** | **CKM syndrome stage 4b onset age (years)^a^** | | | | ***P* for trend** |
| --- | --- | --- | --- | --- | --- | --- | --- | --- |
|  |  |  |  | **<45** | **45-54** | **55-64** | **≥65** |  |
| No. of participants | 1156 | 1156 | / | 26 | 125 | 353 | 652 | / |
| Age, years | 66.12 ± 10.26 | 66.12 ± 10.26 | - | 36.29 ± 11.63 | 51.38 ± 2.62 | 60.56 ± 2.74 | 73.16 ± 5.72 | - |
| Men, n (%) | 972 (84.08) | 972 (84.08) | - | 26 (100.00) | 106 (84.80) | 289 (81.87) | 551 (84.51) | 0.096 |
| Current drinkers, n (%) | 281 (25.62) | 210 (18.63) | <0.001 | 10 (38.46) | 45 (36.00) | 64 (18.50) | 91 (14.44) | <0.001 |
| Current smokers, n (%) | 285 (24.65) | 241 (20.85) | 0.029 | 13 (50.00) | 50 (40.00) | 81 (22.95) | 97 (14.88) | <0.001 |
| Physical activity, n (%) | 217 (19.48) | 252 (22.07) | 0.130 | 4 (15.38) | 24 (19.20) | 75 (21.49) | 149 (23.21) | 0.609 |
| High school or above, n (%) | 152 (13.15) | 121 (10.47) | 0.046 | 5 (19.23) | 25 (20.00) | 30 (8.50) | 61 (9.36) | <0.001 |
| Mental work, n (%) | 180 (15.86) | 206 (17.85) | 0.445 | 5 (19.23) | 18 (14.40) | 53 (15.01) | 130 (20.00) | 0.150 |
| WC, cm | 88.05 ± 10.27 | 90.72 ± 10.34 | <0.001 | 95.50 ± 12.27 | 91.33 ± 9.56 | 91.08 ± 9.90 | 90.22 ± 10.59 | 0.046 |
| BMI, kg/m^2^ | 24.83 ± 3.24 | 26.16 ± 3.74 | <0.001 | 27.55 ± 4.71 | 26.53 ± 4.04 | 26.49 ± 3.55 | 25.85 ± 3.71 | 0.007 |
| SBP, mmHg | 141.22 ± 20.77 | 150.27 ± 22.18 | <0.001 | 137.60 ± 19.46 | 136.64 ± 22.96 | 144.42 ± 21.33 | 148.53 ± 21.58 | <0.001 |
| DBP, mmHg | 83.20 ± 10.94 | 87.95 ± 13.08 | <0.001 | 91.94 ± 13.99 | 90.74 ± 12.81 | 90.96 ± 11.93 | 85.81 ± 12.01 | <0.001 |
| FBG, mmol/L | 6.04 ± 1.96 | 7.13 ± 3.16 | <0.001 | 6.84 ± 2.67 | 7.28 ± 3.46 | 7.47 ± 2.77 | 6.92 ± 2.46 | 0.063 |
| LDL-C, mmol/L | 2.83 ± 1.10 | 2.87 ± 1.02 | 0.233 | 2.56 ± 1.01 | 2.86 ± 0.95 | 2.83 ± 0.99 | 2.87 ± 1.12 | 0.201 |
| HDL-C, mmol/L | 1.48 ± 0.47 | 1.36 ± 0.47 | <0.001 | 1.34 ± 0.39 | 1.42 ± 0.46 | 1.41 ± 0.49 | 1.43 ± 0.48 | 0.570 |
| TG, mmol/L | 1.20 (0.87, 1.82) | 1.51 (1.08, 2.33) | <0.001 | 1.98 (1.46, 3.40) | 1.86 (1.19, 3.00) | 1.57 (1.14, 2.49) | 1.42 (0.93, 2.11) | <0.001 |
| TC, mmol/L | 5.07 ± 1.17 | 5.04 ± 1.43 | 0.614 | 5.86 ± 1.57 | 5.26 ± 1.49 | 5.15 ± 1.34 | 4.93 ± 1.46 | 0.007 |
| hs-CRP, mg/L | 1.60 (0.67, 3.00) | 2.40 (0.97, 4.91) | <0.001 | 2.00 (1.20, 6.20) | 2.30 (0.80, 4.80) | 1.90 (0.90, 4.40) | 2.80 (1.00, 5.30) | 0.045 |
| eGFR, mL/min/1.73m^2^ | 82.89 ± 18.87 | 61.96 ± 25.94 | <0.001 | 37.19 ± 25.38 | 31.01 ± 18.20 | 34.50 ± 17.80 | 34.83 ± 16.17 | 0.022 |
| Family history of CVD, n (%) | 13 (1.16) | 46 (3.98) | <0.001 | 3 (11.54) | 10 (8.00) | 16 (4.55) | 17 (2.61) | 0.005 |
| MetS, n (%) | 450 (38.93) | 710 (61.42) | <0.001 | 16 (61.54) | 79 (63.20) | 228 (64.59) | 387 (59.36) | 0.418 |
| Hypertension, n (%) | 692 (59.86) | 935 (80.88) | <0.001 | 21 (80.77) | 83 (66.40) | 288 (81.59) | 543 (83.28) | <0.001 |
| Diabetes, n (%) | 194 (16.78) | 441 (38.15) | <0.001 | 7 (26.92) | 41 (32.80) | 154 (43.63) | 239 (36.66) | 0.046 |
| Dyslipidemia, n (%) | 736 (63.67) | 829 (71.71) | <0.001 | 22 (84.62) | 91 (72.80) | 97 (72.52) | 192 (70.55) | 0.431 |
| Anti-hypertension drugs, n (%) | 133 (11.51) | 361 (31.23) | <0.001 | 8 (30.77) | 34 (27.20) | 110 (31.16) | 209 (32.06) | 0.764 |
| Hypoglycemic drugs, n (%) | 56 (4.90) | 205 (17.73) | <0.001 | 4 (15.38) | 17 (13.60) | 72 (20.40) | 112 (17.18) | 0.332 |
| Lipid-lowering drugs, n (%) | 6 (0.53) | 24 (2.08) | 0.001 | 1 (3.85) | 3 (2.40) | 8 (2.27) | 12 (1.84) | 0.872 |

Note: Data are presented as mean ± SD, median (interquartile range), or n (%).

BMI, body mass index; CVD, cardiovascular disease; CKD, chronic kidney disease; DBP, diastolic blood pressure; eGFR, estimated glomerular filtration rate; FBG, fasting blood glucose; HDL-C, high-density lipoprotein cholesterol; hs-CRP, high-sensitivity C reactive protein; LDL-C, low-density lipoprotein cholesterol; MetS, metabolic syndrome; SBP, systolic blood pressure; SD, standard deviation; TC, total cholesterol; TG, triglycerides; WC, waist circumference.

^a^ Basic characteristics for new-onset CKM syndrome stage 4b participants across age groups.

**Table S8. Age-Stratified Mortality Dynamics in Incident Advanced CKM Syndrome: Event Rates, Absolute Risk Differences, and Hazard Ratios**

| **Age Group(years)** | **<45** | **45-54** | **55-64** | **≥65** |
| --- | --- | --- | --- | --- |
| **Control Event Rate (/1000 PY; Events/Persons)** | 0.69 (8/1,336) | 4.36 (101/2,319) | 7.16 (487/6,803) | 23.22 (1,701/7,325) |
| **Incident Case Event Rate (/1000 PY; Events/Persons)** | 2.18 (29/1,336) | 10.78 (250/2,319) | 13.77 (937/6,803) | 27.77 (2,034/7,325) |
| **Absolute Risk Difference(ΔRate) (/1000 PY; Incident - Control)** | 1.49 | 6.42 | 8.61 | 4.55 |
| **Hazard ratio (95%CI)** | 3.35 (1.52-7.38) | 2.58 (2.04-3.26) | 2.00 (1.79-2.23) | 1.17 (1.09-1.25) |
| **P value** | 0.003 | <0.001 | <0.001 | <0.001 |
| **% Reduction(vs. Previous Group)** |  | 22.99% | 22.48% | 41.50% |

Note: Poisson regression for ΔRate calculations (offset = log person-time); Cox proportional hazards models stratified by age group with adjustment for: Behavioral: Smoking status, alcohol intake; Metabolic: Systolic BP, lipid profiles, BMI

Socioeconomic: Education, occupation; Interaction P-value: Wald test for HR heterogeneity across age strata (P<sub>interaction</sub> <0.001). Event rates: Expressed per 1,000 person-years (PY); values indicate events/total persons; Absolute Risk Difference (ΔRate): Incident case rate minus control rate, per 1,000 PY; Hazard Ratio (95% CI): Derived from multivariable Cox models, reference = matched controls; % Reduction: Decline in HR relative to the adjacent younger age stratum.

**Table S9.** Age-Stratified Mortality Risk Assessment: Adjusted Hazard Ratios for Baseline Covariates in Advanced CKM Syndrome versus Controls

| **Variables** | **No.** | **Hazard ratio (95% CI)** | | | | | | | | ***P* for interaction** |
| --- | --- | --- | --- | --- | --- | --- | --- | --- | --- | --- |
|  |  | **Onset age (years)<45** | *P* value | **45-54** | *P* value | **55-64** | *P* value | **≥65** | *P* value |  |
| **Smoking status** |  |  |  |  |  |  |  |  |  |  |
| Noncurrent smokers | 23807 | 2.53 (0.85-7.49) | 0.094 | 2.58 (1.84-3.62) | <0.001 | 2.09 (1.82-2.40) | <0.001 | 1.15 (1.07-1.24) | <0.001 | <0.001 |
| Current smokers | 10759 | 5.27 (1.54-17.98) | 0.008 | 2.56 (1.86-3.53) | <0.001 | 1.82 (1.52-2.19) | <0.001 | 1.22 (1.06-1.40) | 0.005 | <0.001 |
| **Drinking status** |  |  |  |  |  |  |  |  |  |  |
| Noncurrent drinkers | 24796 | 4.07 (1.49-11.13) | 0.006 | 2.71 (1.99-3.67) | <0.001 | 2.14 (1.88-2.44) | <0.001 | 1.18 (1.10-1.27) | <0.001 | <0.001 |
| Current drinkers | 9770 | 2.71 (0.75-9.81) | 0.129 | 2.40 (1.67-3.44) | <0.001 | 1.65 (1.34-2.03) | <0.001 | 1.14 (0.99-1.33) | 0.079 | <0.001 |
| **Smoking & drinking status** |  |  |  |  |  |  |  |  |  |  |
| Current nonsmoking & nondrinking | 19437 | 3.19 (0.97-10.45) | 0.055 | 2.53 (1.75-3.67) | <0.001 | 2.21 (1.90-2.57) | <0.001 | 1.17 (1.08-1.26) | <0.001 | <0.001 |
| Current smoking and/or drinking | 15129 | 4.17 (1.40-12.40) | 0.010 | 2.61 (1.93-3.52) | <0.001 | 1.77 (1.51-2.09) | <0.001 | 1.19 (1.06-1.34) | 0.004 | <0.001 |
| **Hypertension** |  |  |  |  |  |  |  |  |  |  |
| Without | 13305 | 2.48 (0.85-7.26) | 0.096 | 2.46 (1.67-3.63) | <0.001 | 2.24 (1.83-2.75) | <0.001 | 1.17 (1.03-1.34) | 0.017 | <0.001 |
| With | 21261 | 3.69 (1.07-12.74) | 0.039 | 2.25 (1.66-3.05) | <0.001 | 1.80 (1.57-2.07) | <0.001 | 1.11 (1.02-1.21) | 0.014 | <0.001 |
| **Diabetes** |  |  |  |  |  |  |  |  |  |  |
| Without | 27588 | 3.16 (1.34-7.44) | 0.009 | 2.38 (1.81-3.12) | <0.001 | 1.85 (1.63-2.11) | <0.001 | 1.14 (1.06-1.23) | <0.001 | <0.001 |
| With | 6978 | 4.96 (0.56-44.12) | 0.151 | 2.29 (1.41-3.72) | <0.001 | 1.62 (1.25-2.08) | <0.001 | 0.90 (0.73-1.11) | 0.331 | <0.001 |
| **Dyslipidemia** |  |  |  |  |  |  |  |  |  |  |
| Without | 11862 | 1.60 (0.44-5.81) | 0.473 | 2.30 (1.50-3.51) | <0.001 | 1.80 (1.49-2.17) | <0.001 | 1.20 (1.08-1.32) | <0.001 | <0.001 |
| With | 22704 | 4.97 (1.70-14.53) | 0.003 | 2.62 (1.97-3.47) | <0.001 | 2.14 (1.86-2.46) | <0.001 | 1.18 (1.08-1.29) | <0.001 | <0.001 |
| **MetS** |  |  |  |  |  |  |  |  |  |  |
| Without | 19508 | 1.66 (0.63-4.34) | 0.305 | 2.20 (1.59-3.03) | <0.001 | 1.90 (1.63-2.22) | <0.001 | 1.10 (1.01-1.20) | 0.033 | <0.001 |
| With | 15058 | 12.89 (1.71-96.96) | 0.013 | 2.66 (1.84-3.86) | <0.001 | 2.03 (1.70-2.43) | <0.001 | 1.24 (1.10-1.40) | <0.001 | <0.001 |
| **hs-CRP levels** |  |  |  |  |  |  |  |  |  |  |
| <3 mg/L | 22694 | 2.02 (0.73-5.62) | 0.178 | 2.11 (1.57-2.83) | <0.001 | 1.81 (1.57-2.09) | <0.001 | 1.14 (1.04-1.25) | 0.004 | <0.001 |
| ≥3 mg/L | 11872 | 10.15 (1.32-77.85) | 0.026 | 3.32 (2.18-5.05) | <0.001 | 2.26 (1.87-2.73) | <0.001 | 1.18 (1.06-1.30) | 0.002 | <0.001 |
| **HDL-C levels** |  |  |  |  |  |  |  |  |  |  |
| <1.3 mmol/L | 16183 | 17.94 (2.37-136.01) | 0.005 | 2.14 (1.51-3.05) | <0.001 | 1.90 (1.61-2.24) | <0.001 | 1.20 (1.08-1.34) | 0.001 | <0.001 |
| ≥1.3 mmol/L | 18383 | 2.76 (1.15-6.62) | 0.023 | 2.54 (1.97-3.27) | <0.001 | 2.01 (1.78-2.27) | <0.001 | 1.13 (1.05-1.22) | <0.001 | <0.001 |

Note: The model was adjusted for smoking status, drinking status, physical activity, education, work type, and high-sensitivity C-reactive protein. No., number of participants, including case subjects and control subjects; *P* for interaction, the interaction between cases and their ages at diagnosis.

**Table S10. Subgroup analysis (based on** **smoking status) of the hazard ratios (95% CI) for all-cause mortality among** **participants with new-onset cases versus control subjects across age-groups.**

|  | **Non-current smokers** | | **Current smokers** | |
| --- | --- | --- | --- | --- |
| **Onset age (years)** | **HR (95%CI)** | ***P* value** | **HR (95%CI)** | ***P* value** |
| **New-onset advanced CKM syndrome** | | | | |
| **N** | 23807 | | 10759 | |
| **<45** | 2.53 (0.85-7.49) | 0.094 | 5.27 (1.54-17.98) | 0.008 |
| **45-54** | 2.58 (1.84-3.62) | <0.001 | 2.56 (1.86-3.53) | <0.001 |
| **55-64** | 2.09 (1.82-2.40) | <0.001 | 1.82 (1.52-2.19) | <0.001 |
| **≥65** | 1.15 (1.07-1.24) | <0.001 | 1.22 (1.06-1.40) | 0.005 |
| ***P* for interaction** | <0.001 | | <0.001 | |
| **New-onset CKM syndrome stage 3** | | | | |
| **N** | 15719 | | 7013 | |
| **<45** | 4.13 (0.82-20.88) | 0.087 | 4.15 (0.50-34.21) | 0.186 |
| **45-54** | 2.72 (1.54-4.81) | <0.001 | 3.23 (1.86-5.60) | <0.001 |
| **55-64** | 1.78 (1.47-2.16) | <0.001 | 1.89 (1.44-2.48) | <0.001 |
| **≥65** | 0.99 (0.90-1.08) | 0.742 | 1.11 (0.95-1.31) | 0.197 |
| ***P* for interaction** | <0.001 | | <0.001 | |
| **New-onset CKM syndrome stage 4** | | | | |
| **N** | 11380 | | 4568 | |
| **<45** | 4.91 (0.55-44.11) | 0.156 | 4.76 (1.05-21.69) | 0.044 |
| **45-54** | 2.70 (1.77-4.10) | <0.001 | 2.71 (1.82-4.04) | <0.001 |
| **55-64** | 2.13 (1.78-2.56) | <0.001 | 2.08 (1.61-2.70) | <0.001 |
| **≥65** | 1.72 (1.56-1.90) | <0.001 | 1.83 (1.49-2.25) | <0.001 |
| ***P* for interaction** | <0.001 | | 0.019 | |
| **New-onset CKM syndrome stage 4a** | | | | |
| **N** | 10616 | | 4384 | |
| **<45** | 3.61 (0.37-34.84) | 0.268 | 4.80 (1.03-22.34) | 0.045 |
| **45-54** | 2.35 (1.52-3.65) | <0.001 | 2.43 (1.62-3.63) | <0.001 |
| **55-64** | 2.04 (1.68-2.47) | <0.001 | 1.87 (1.43-2.44) | <0.001 |
| **≥65** | 1.64 (1.47-1.82) | <0.001 | 1.73 (1.40-2.15) | <0.001 |
| ***P* for interaction** | <0.001 | | 0.056 | |
| **New-onset CKM syndrome stage 4b** | | | | |
| **N** | 1786 | | 526 | |
| **<45** | NA | NA | 5.25 (0.59-47.09) | 0.139 |
| **45-54** | 10.61 (3.16-35.68) | <0.001 | 2.74 (0.88-8.54) | 0.082 |
| **55-64** | 3.47 (2.21-5.46) | <0.001 | 3.06 (1.63-5.77) | <0.001 |
| **≥65** | 2.29 (1.88-2.78) | <0.001 | 2.66 (1.70-4.16) | <0.001 |
| ***P* for interaction** | <0.001 | | 0.394 | |

Note: The model was adjusted for smoking status, drinking status, physical activity, education, work type, and high-sensitivity C-reactive protein.

N, number of participants, including case subjects and control subjects; *P* for interaction, the interaction between cases and their ages at diagnosis.

**Table S11. Subgroup analysis (based on drinking status) of the hazard ratios (95% CI) for all-cause mortality among participants with new-onset cases versus control subjects across age-groups.**

|  | **Non-current drinkers** | | **Current drinkers** | |
| --- | --- | --- | --- | --- |
| **Onset age (years)** | **HR (95%CI)** | ***P* value** | **HR (95%CI)** | ***P* value** |
| **New-onset advanced CKM syndrome** | | | | |
| **N** | 24796 | | 9770 | |
| **<45** | 4.07 (1.49-11.13) | 0.006 | 2.71 (0.75-9.81) | 0.129 |
| **45-54** | 2.71 (1.99-3.67) | <0.001 | 2.40 (1.67-3.44) | <0.001 |
| **55-64** | 2.14 (1.88-2.44) | <0.001 | 1.65 (1.34-2.03) | <0.001 |
| **≥65** | 1.18 (1.10-1.27) | <0.001 | 1.14 (0.99-1.33) | 0.079 |
| ***P* for interaction** | <0.001 | | <0.001 | |
| **New-onset CKM syndrome stage 3** | | | | |
| **N** | 16329 | | 6403 | |
| **<45** | 5.43 (1.11-26.54) | 0.037 | 5.88 (0.66-63.06) | 0.143 |
| **45-54** | 3.63 (2.14-6.15) | <0.001 | 2.33 (1.30-4.19) | 0.005 |
| **55-64** | 1.86 (1.54-2.25) | <0.001 | 1.68 (1.26-2.24) | <0.001 |
| **≥65** | 1.04 (0.96-1.13) | 0.397 | 0.94 (0.80-1.11) | 0.470 |
| ***P* for interaction** | <0.001 | | <0.001 | |
| **New-onset CKM syndrome stage 4** | | | | |
| **N** | 11689 | | 4259 | |
| **<45** | 9.03 (1.14-71.56) | 0.037 | 3.03 (0.60-15.38) | 0.181 |
| **45-54** | 2.66 (1.83-3.89) | <0.001 | 2.79 (1.77-4.41) | <0.001 |
| **55-64** | 2.28 (1.92-2.72) | <0.001 | 1.73 (1.30-2.29) | <0.001 |
| **≥65** | 1.77 (1.60-1.95) | <0.001 | 1.62 (1.30-2.00) | <0.001 |
| ***P* for interaction** | <0.001 | | 0.047 | |
| **New-onset CKM syndrome stage 4a** | | | | |
| **N** | 10942 | | 4058 | |
| **<45** | 6.73 (0.82-55.41) | 0.076 | 3.22 (0.63-16.41) | 0.159 |
| **45-54** | 2.20 (1.50-3.24) | <0.001 | 2.69 (1.69-4.26) | <0.001 |
| **55-64** | 2.03 (1.70-2.43) | <0.001 | 1.82 (1.34-2.47) | <0.001 |
| **≥65** | 1.70 (1.53-1.88) | <0.001 | 1.49 (1.19-1.86) | <0.001 |
| ***P* for interaction** | 0.009 | | 0.015 | |
| **New-onset CKM syndrome stage 4b** | | | | |
| **N** | 1821 | | 491 | |
| **<45** | NA | NA | NA | NA |
| **45-54** | 15.62 (3.66-66.77) | <0.001 | 1.92 (0.45-8.30) | 0.382 |
| **55-64** | 3.21 (2.13-4.83) | <0.001 | 3.98 (1.76-8.97) | <0.001 |
| **≥65** | 2.21 (1.83-2.68) | <0.001 | 3.01 (1.84-4.95) | <0.001 |
| ***P* for interaction** | <0.001 | | 0.596 | |

Note: The model was adjusted for smoking status, drinking status, physical activity, education, work type, and high-sensitivity C-reactive protein.

N, number of participants, including case subjects and control subjects; *P* for interaction, the interaction between cases and their ages at diagnosis.

**Table S12. Subgroup analysis (based on smoking status and drinking status) of the hazard ratios (95% CI) for all-cause mortality among participants with new-onset cases versus control subjects across age-groups.**

|  | **Participants without current smoking or current drinking** | | **Participants with current smoking and/or current drinking** | |
| --- | --- | --- | --- | --- |
| **Onset age (years)** | **HR (95%CI)** | ***P* value** | **HR (95%CI)** | ***P* value** |
| **New-onset advanced CKM syndrome** | | | | |
| **N** | 19437 | | 15129 | |
| **<45** | 3.19 (0.97-10.45) | 0.055 | 4.17 (1.40-12.40) | 0.010 |
| **45-54** | 2.53 (1.75-3.67) | <0.001 | 2.61 (1.93-3.52) | <0.001 |
| **55-64** | 2.21 (1.90-2.57) | <0.001 | 1.77 (1.51-2.09) | <0.001 |
| **≥65** | 1.17 (1.08-1.26) | <0.001 | 1.19 (1.06-1.34) | 0.004 |
| ***P* for interaction** | <0.001 | | <0.001 | |
| **New-onset CKM syndrome stage 3** | | | | |
| **N** | 12749 | | 9983 | |
| **<45** | 9.76 (1.07-88.94) | 0.043 | 3.57 (0.67-19.16) | 0.138 |
| **45-54** | 3.09 (1.67-5.74) | <0.001 | 2.84 (1.72-2.19) | <0.001 |
| **55-64** | 1.88 (1.52-2.34) | <0.001 | 1.74 (1.38-2.19) | <0.001 |
| **≥65** | 1.02 (0.93-1.11) | 0.749 | 1.05 (0.92-1.20) | 0.463 |
| ***P* for interaction** | <0.001 | | <0.001 | |
| **New-onset CKM syndrome stage 4** | | | | |
| **N** | 9485 | | 6463 | |
| **<45** | 4.92 (0.55-44.26) | 0.155 | 4.81 (1.06-21.88) | 0.042 |
| **45-54** | 2.61 (1.64-4.17) | <0.001 | 2.77 (1.91-3.99) | <0.001 |
| **55-64** | 2.17 (1.79-2.64) | <0.001 | 2.05 (1.62-2.58) | <0.001 |
| **≥65** | 1.71 (1.54-1.89) | <0.001 | 1.81 (1.52-2.15) | <0.001 |
| ***P* for interaction** | 0.003 | | 0.009 | |
| **New-onset CKM syndrome stage 4a** | | | | |
| **N** | 8826 | | 6174 | |
| **<45** | 3.65 (0.37-35.25) | 0.264 | 4.85 (1.04-22.51) | 0.044 |
| **45-54** | 2.17 (1.33-3.52) | 0.002 | 2.53 (1.74-3.68) | <0.001 |
| **55-64** | 2.04 (1.67-2.50) | <0.001 | 1.89 (1.48-2.40) | <0.001 |
| **≥65** | 1.64 (1.47-1.83) | <0.001 | 1.68 (1.40-2.00) | <0.001 |
| ***P* for interaction** | 0.019 | | 0.016 | |
| **New-onset CKM syndrome stage 4b** | | | | |
| **N** | 1535 | | 777 | |
| **<45** | NA | NA | 5.25 (0.59-47.09) | 0.139 |
| **45-54** | 12.30 (2.87-52.71) | <0.001 | 2.85 (1.02-7.98) | 0.047 |
| **55-64** | 3.54 (2.23-5.61) | <0.001 | 3.54 (1.99-6.29) | <0.001 |
| **≥65** | 2.12 (1.74-2.59) | <0.001 | 2.57 (1.80-3.68) | <0.001 |
| ***P* for interaction** | <0.001 | | 0.309 | |

Note: The model was adjusted for smoking status, drinking status, physical activity, education, work type, and high-sensitivity C-reactive protein.

N, number of participants, including case subjects and control subjects; *P* for interaction, the interaction between cases and their ages at diagnosis.

**Table S13. Subgroup analysis (based on hypertension) of the hazard ratios (95% CI) for** **all-cause mortality among participants with new-onset cases versus control subjects across age-groups.**

|  | **Participants without hypertension** | | **Participants with hypertension** | |
| --- | --- | --- | --- | --- |
| **Onset age (years)** | **HR (95%CI)** | ***P* value** | **HR (95%CI)** | ***P* value** |
| **New-onset advanced CKM syndrome** | | | | |
| **N** | 13305 | | 21261 | |
| **<45** | 2.48 (0.85-7.26) | 0.096 | 3.69 (1.07-12.74) | 0.039 |
| **45-54** | 2.46 (1.67-3.63) | <0.001 | 2.25 (1.66-3.05) | <0.001 |
| **55-64** | 2.24 (1.83-2.75) | <0.001 | 1.80 (1.57-2.07) | <0.001 |
| **≥65** | 1.17 (1.03-1.34) | 0.017 | 1.11 (1.02-1.21) | 0.014 |
| ***P* for interaction** | <0.001 | | <0.001 | |
| **New-onset CKM syndrome stage 3** | | | | |
| **N** | 8172 | | 14560 | |
| **<45** | 3.64 (0.50-14.01) | 0.254 | 6.02 (0.76-50.92) | 0.089 |
| **45-54** | 3.27 (1.64-6.53) | <0.001 | 2.93 (1.68-5.09) | <0.001 |
| **55-64** | 1.77 (1.12-2.79) | 0.014 | 1.69 (1.38-2.06) | <0.001 |
| **≥65** | 1.00 (0.83-1.14) | 0.690 | 0.98 (0.90-1.04) | 0.235 |
| ***P* for interaction** | <0.001 | | <0.001 | |
| **New-onset CKM syndrome stage 4** | | | | |
| **N** | 6029 | | 9919 | |
| **<45** | 6.36 (0.75-54.19) | 0.091 | 3.27 (0.70-15.31) | 0.132 |
| **45-54** | 2.63 (1.60-4.31) | <0.001 | 2.36 (1.64-3.39) | <0.001 |
| **55-64** | 1.98 (1.54-2.55) | <0.001 | 2.07 (1.72-2.50) | <0.001 |
| **≥65** | 1.75 (1.49-2.06) | <0.001 | 1.71 (1.54-1.91) | <0.001 |
| ***P* for interaction** | 0.040 | | 0.005 | |
| **New-onset CKM syndrome stage 4a** | | | | |
| **N** | 5795 | | 9205 | |
| **<45** | 6.64 (0.78-56.33) | 0.083 | 3.03 (0.59-15.63) | 0.186 |
| **45-54** | 2.17 (1.30-3.61) | 0.003 | 2.15 (1.48-3.12) | <0.001 |
| **55-64** | 2.02 (1.55-2.62) | <0.001 | 1.85 (1.53-2.25) | <0.001 |
| **≥65** | 1.69 (1.43-2.00) | <0.001 | 1.61 (1.44-1.81) | <0.001 |
| ***P* for interaction** | 0.081 | | 0.027 | |
| **New-onset CKM syndrome stage 4b** | | | | |
| **N** | 685 | | 1627 | |
| **<45** | NA | NA | 4.61 (0.49-42.62) | 0.183 |
| **45-54** | 4.83 (1.69-13.81) | 0.003 | 10.27 (2.37-44.50) | 0.002 |
| **55-64** | 2.48 (1.33-4.63) | 0.004 | 4.36 (2.20-7.20) | <0.001 |
| **≥65** | 2.42 (1.90-3.78) | <0.001 | 2.05 (1.68-2.50) | <0.001 |
| ***P* for interaction** | 0.525 | | <0.001 | |

Note: The model was adjusted for smoking status, drinking status, physical activity, education, work type, and high-sensitivity C-reactive protein.

N, number of participants, including case subjects and control subjects; *P* for interaction, the interaction between cases and their ages at diagnosis.

**Table S14. Subgroup analysis (based on diabetes) of the hazard ratios (95% CI) for all-cause mortality among participants with new-onset cases versus control subjects across age-groups.**

|  | **Participants without diabetes** | | **Participants with diabetes** | |
| --- | --- | --- | --- | --- |
| **Onset age (years)** | **HR (95%CI)** | ***P* value** | **HR (95%CI)** | ***P* value** |
| **New-onset advanced CKM syndrome** | | | | |
| **N** | 27588 | | 6978 | |
| **<45** | 3.16 (1.34-7.44) | 0.009 | 4.96 (0.56-44.12) | 0.151 |
| **45-54** | 2.38 (1.81-3.12) | <0.001 | 2.29 (1.41-3.72) | <0.001 |
| **55-64** | 1.85 (1.63-2.11) | <0.001 | 1.62 (1.25-2.08) | <0.001 |
| **≥65** | 1.14 (1.06-1.23) | <0.001 | 0.90 (0.73-1.11) | 0.331 |
| ***P* for interaction** | <0.001 | | <0.001 | |
| **New-onset CKM syndrome stage 3** | | | | |
| **N** | 17631 | | 5101 | |
| **<45** | 4.06 (1.10-15.06) | 0.036 | NA | NA |
| **45-54** | 2.71 (1.67-4.40) | <0.001 | 1.81 (0.88-3.73) | 0.107 |
| **55-64** | 1.61 (1.32-1.97) | <0.001 | 1.48 (1.02-2.14) | 0.037 |
| **≥65** | 0.98 (0.90-1.06) | 0.247 | 0.86 (0.58-1.14) | 0.124 |
| ***P* for interaction** | <0.001 | | <0.001 | |
| **New-onset CKM syndrome stage 4** | | | | |
| **N** | 12872 | | 3076 | |
| **<45** | 3.88 (1.08-13.96) | 0.038 | NA | NA |
| **45-54** | 2.70 (1.94-3.78) | <0.001 | 2.21 (1.23-3.96) | 0.008 |
| **55-64** | 1.99 (1.67-2.37) | <0.001 | 2.06 (1.54-2.75) | <0.001 |
| **≥65** | 1.68 (1.52-1.86) | <0.001 | 1.80 (1.48-2.18) | <0.001 |
| ***P* for interaction** | <0.001 | | 0.195 | |
| **New-onset CKM syndrome stage 4a** | | | | |
| **N** | 12264 | | 2736 | |
| **<45** | 3.50 (0.94-13.02) | 0.061 | NA | NA |
| **45-54** | 2.42 (1.72-3.41) | <0.001 | 1.97 (1.08-3.58) | 0.027 |
| **55-64** | 1.91 (1.60-2.29) | <0.001 | 1.82 (1.33-2.50) | <0.001 |
| **≥65** | 1.62 (1.46-1.80) | <0.001 | 1.64 (1.33-2.02) | <0.001 |
| ***P* for interaction** | 0.003 | | 0.288 | |
| **New-onset CKM syndrome stage 4b** | | | | |
| **N** | 1677 | | 635 | |
| **<45** | 5.42 (0.60-48.80) | 0.132 | NA | NA |
| **45-54** | 6.92 (2.35-20.38) | <0.001 | 3.24 (0.59-17.86) | 0.177 |
| **55-64** | 2.59 (1.64-4.09) | <0.001 | 3.02 (1.49-6.12) | 0.002 |
| **≥65** | 2.19 (1.78-2.70) | <0.001 | 2.44 (1.69-3.53) | <0.001 |
| ***P* for interaction** | 0.003 | | 0.650 | |

Note: The model was adjusted for smoking status, drinking status, physical activity, education, work type, and high-sensitivity C-reactive protein.

N, number of participants, including case subjects and control subjects; *P* for interaction, the interaction between cases and their ages at diagnosis.

**Table S15. Subgroup analysis (based on dyslipidemia) of the hazard ratios (95% CI) for all-cause mortality among participants with new-onset cases versus control subjects across age-groups.**

|  | **Participants without dyslipidemia** | | **Participants with dyslipidemia** | |
| --- | --- | --- | --- | --- |
| **Onset age (years)** | **HR (95%CI)** | ***P* value** | **HR (95%CI)** | ***P* value** |
| **New-onset advanced CKM syndrome** | | | | |
| **N** | 11862 | | 22704 | |
| **<45** | 1.60 (0.44-5.81) | 0.473 | 4.97 (1.70-14.53) | 0.003 |
| **45-54** | 2.30 (1.50-3.51) | <0.001 | 2.62 (1.97-3.47) | <0.001 |
| **55-64** | 1.80 (1.49-2.17) | <0.001 | 2.14 (1.86-2.46) | <0.001 |
| **≥65** | 1.20 (1.08-1.32) | <0.001 | 1.18 (1.08-1.29) | <0.001 |
| ***P* for interaction** | <0.001 | | <0.001 | |
| **New-onset CKM syndrome stage 3** | | | | |
| **N** | 7829 | | 14903 | |
| **<45** | 3.81 (0.39-37.06) | 0.249 | 4.99 (1.07-23.26) | 0.041 |
| **45-54** | 4.59 (2.06-10.26) | <0.001 | 2.53 (1.62-3.96) | <0.001 |
| **55-64** | 1.65 (1.21-2.26) | 0.002 | 1.82 (1.50-2.20) | <0.001 |
| **≥65** | 1.03 (0.91-1.16) | 0.661 | 1.03 (0.93-1.14) | 0.590 |
| ***P* for interaction** | <0.001 | | <0.001 | |
| **New-onset CKM syndrome stage 4** | | | | |
| **N** | 5530 | | 10418 | |
| **<45** | 2.54 (0.19-33.31) | 0.479 | 4.99 (1.12-22.21) | 0.035 |
| **45-54** | 2.08 (1.26-3.43) | 0.004 | 2.96 (2.06-4.24) | <0.001 |
| **55-64** | 1.97 (1.55-2.52) | <0.001 | 2.20 (1.82-2.66) | <0.001 |
| **≥65** | 1.76 (1.53-2.03) | <0.001 | 1.71 (1.53-1.93) | <0.001 |
| ***P* for interaction** | 0.260 | | <0.001 | |
| **New-onset CKM syndrome stage 4a** | | | | |
| **N** | 5235 | | 9765 | |
| **<45** | 2.11 (0.17-26.83) | 0.565 | 5.11 (1.11-23.48) | 0.036 |
| **45-54** | 1.89 (1.10-3.26) | 0.021 | 2.49 (1.74-3.56) | <0.001 |
| **55-64** | 2.10 (1.63-2.75) | <0.001 | 1.89 (1.56-2.28) | <0.001 |
| **≥65** | 1.68 (1.45-1.94) | <0.001 | 1.63 (1.44-1.84) | <0.001 |
| ***P* for interaction** | 0.155 | | 0.004 | |
| **New-onset CKM syndrome stage 4b** | | | | |
| **N** | 747 | | 1565 | |
| **<45** | NA | NA | 9.53 (0.41-30.40) | 0.250 |
| **45-54** | 7.13 (1.82-27.86) | 0.005 | 6.58 (1.91-22.71) | 0.003 |
| **55-64** | 1.96 (1.11-3.48) | 0.021 | 4.91 (2.95-8.19) | <0.001 |
| **≥65** | 1.96 (1.49-2.69) | <0.001 | 2.58 (2.05-3.26) | <0.001 |
| ***P* for interaction** | 0.118 | | 0.002 | |

Note: The model was adjusted for smoking status, drinking status, physical activity, education, work type, and high-sensitivity C-reactive protein.

N, number of participants, including case subjects and control subjects; *P* for interaction, the interaction between cases and their ages at diagnosis.

**Table S16. Subgroup analysis (based on MetS) of the hazard ratios (95% CI) for all-cause mortality among participants with new-onset cases versus control subjects across age-groups.**

|  | **Participants without MetS** | | **Participants with MetS** | |
| --- | --- | --- | --- | --- |
| **Onset age (years)** | **HR (95%CI)** | ***P* value** | **HR (95%CI)** | ***P* value** |
| **New-onset advanced CKM syndrome** | | | | |
| **N** | 19508 | | 15058 | |
| **<45** | 1.66 (0.63-4.34) | 0.305 | 12.89 (1.71-96.96) | 0.013 |
| **45-54** | 2.20 (1.59-3.03) | <0.001 | 2.66 (1.84-3.86) | <0.001 |
| **55-64** | 1.90 (1.63-2.22) | <0.001 | 2.03 (1.70-2.43) | <0.001 |
| **≥65** | 1.10 (1.01-1.20) | 0.033 | 1.24 (1.10-1.40) | <0.001 |
| ***P* for interaction** | <0.001 | | <0.001 | |
| **New-onset CKM syndrome stage 3** | | | | |
| **N** | 12944 | | 9788 | |
| **<45** | 3.58 (0.64-20.19) | 0.148 | 7.30 (0.90-59.30) | 0.063 |
| **45-54** | 3.13 (1.80-5.45) | <0.001 | 2.68 (1.47-4.89) | 0.001 |
| **55-64** | 1.77 (1.40-2.24) | <0.001 | 1.71 (1.34-2.18) | <0.001 |
| **≥65** | 0.98 (0.89-1.09) | 0.739 | 1.07 (0.94-1.23) | 0.314 |
| ***P* for interaction** | <0.001 | | <0.001 | |
| **New-onset CKM syndrome stage 4** | | | | |
| **N** | 8709 | | 7239 | |
| **<45** | 1.76 (0.42-7.35) | 0.436 | NA | NA |
| **45-54** | 2.35 (1.58-3.49) | <0.001 | 2.77 (1.78-4.29) | <0.001 |
| **55-64** | 1.95 (1.59-2.40) | <0.001 | 2.16 (1.72-2.72) | <0.001 |
| **≥65** | 1.73 (1.54-1.95) | <0.001 | 1.69 (1.47-1.95) | <0.001 |
| ***P* for interaction** | 0.062 | | <0.001 | |
| **New-onset CKM syndrome stage 4a** | | | | |
| **N** | 8341 | | 6659 | |
| **<45** | 1.77 (0.43-7.33) | 0.431 | NA | NA |
| **45-54** | 2.07 (1.38-3.09) | <0.001 | 2.54 (1.61-4.02) | <0.001 |
| **55-64** | 1.81 (1.47-2.22) | <0.001 | 2.05 (1.61-2.62) | <0.001 |
| **≥65** | 1.65 (1.46-1.86) | <0.001 | 1.58 (1.36-1.84) | <0.001 |
| ***P* for interaction** | 0.163 | | 0.002 | |
| **New-onset CKM syndrome stage 4b** | | | | |
| **N** | 1152 | | 1160 | |
| **<45** | NA | NA | NA | NA |
| **45-54** | 4.52 (1.37-14.90) | 0.013 | 6.42 (1.39-29.64) | 0.017 |
| **55-64** | 2.52 (1.51-4.20) | <0.001 | 4.50 (2.44-8.31) | <0.001 |
| **≥65** | 2.18 (1.69-2.80) | <0.001 | 2.22 (1.68-2.92) | <0.001 |
| ***P* for interaction** | 0.071 | | 0.006 | |

Note: The model was adjusted for smoking status, drinking status, physical activity, education, work type, and high-sensitivity C-reactive protein.

N, number of participants, including case subjects and control subjects; *P* for interaction, the interaction between cases and their ages at diagnosis.

**Table S17. Subgroup analysis (based on hs-CRP) of the hazard ratios (95% CI) for all-cause mortality among participants with new-onset cases versus control subjects across age-groups.**

|  | **Participants with hs-CRP<3mg/L** | | **Participants with hs-CRP≥3mg/L** | |
| --- | --- | --- | --- | --- |
| **Onset age (years)** | **HR (95%CI)** | ***P* value** | **HR (95%CI)** | ***P* value** |
| **New-onset advanced CKM syndrome** | | | | |
| **N** | 22694 | | 11872 | |
| **<45** | 2.02 (0.73-5.62) | 0.178 | 10.15 (1.32-77.85) | 0.026 |
| **45-54** | 2.11 (1.57-2.83) | <0.001 | 3.32 (2.18-5.05) | <0.001 |
| **55-64** | 1.81 (1.57-2.09) | <0.001 | 2.26 (1.87-2.73) | <0.001 |
| **≥65** | 1.14 (1.04-1.25) | 0.004 | 1.18 (1.06-1.30) | 0.002 |
| ***P* for interaction** | <0.001 | | <0.001 | |
| **New-onset CKM syndrome stage 3** | | | | |
| **N** | 14795 | | 7937 | |
| **<45** | 2.40 (0.43-13.24) | 0.316 | 6.68 (0.75-59.84) | 0.090 |
| **45-54** | 2.38 (1.48-3.84) | <0.001 | 4.51 (2.12-9.62) | <0.001 |
| **55-64** | 1.72 (1.40-2.13) | <0.001 | 1.78 (1.37-2.30) | <0.001 |
| **≥65** | 1.05 (0.94-1.16) | 0.384 | 0.99 (0.88-1.10) | 0.807 |
| ***P* for interaction** | <0.001 | | <0.001 | |
| **New-onset CKM syndrome stage 4** | | | | |
| **N** | 10222 | | 5726 | |
| **<45** | 3.15 (0.62-15.94) | 0.166 | 7.27 (0.89-59.31) | 0.064 |
| **45-54** | 2.05 (1.42-2.95) | <0.001 | 3.91 (2.33-6.55) | <0.001 |
| **55-64** | 2.00 (1.65-2.43) | <0.001 | 2.19 (1.72-2.78) | <0.001 |
| **≥65** | 1.67 (1.47-1.89) | <0.001 | 1.78 (1.56-2.03) | <0.001 |
| ***P* for interaction** | 0.046 | | <0.001 | |
| **New-onset CKM syndrome stage 4a** | | | | |
| **N** | 9707 | | 5293 | |
| **<45** | 2.81 (0.56-14.18) | 0.211 | 7.33 (0.87-62.00) | 0.068 |
| **45-54** | 1.97 (1.34-2.88) | <0.001 | 3.05 (1.84-5.05) | <0.001 |
| **55-64** | 1.86 (1.52-2.28) | <0.001 | 2.02 (1.58-2.59) | <0.001 |
| **≥65** | 1.61 (1.41-1.83) | <0.001 | 1.64 (1.42-1.89) | <0.001 |
| ***P* for interaction** | 0.078 | | 0.003 | |
| **New-onset CKM syndrome stage 4b** | | | | |
| **N** | 1329 | | 983 | |
| **<45** | NA | NA | NA | NA |
| **45-54** | 4.63 (1.22-17.59) | 0.025 | 4.88 (1.39-17.19) | 0.014 |
| **55-64** | 3.18 (1.94-5.22) | <0.001 | 3.73 (2.08-6.71) | <0.001 |
| **≥65** | 2.28 (1.77-2.93) | <0.001 | 2.20 (1.68-2.88) | <0.001 |
| ***P* for interaction** | 0.095 | | 0.080 | |

Note: The model was adjusted for smoking status, drinking status, physical activity, education, work type, and high-sensitivity C-reactive protein.

N, number of participants, including case subjects and control subjects; *P* for interaction, the interaction between cases and their ages at diagnosis.

**Table S18. Subgroup analysis (based on HDL-C) of the hazard ratios (95% CI) for all-cause mortality among participants with new-onset cases versus control subjects across age-groups.**

|  | **Participants with** **HDL-C<1.3** **mmol/L** | | **Participants with HDL-C≥1.3** **mmol/L** | |
| --- | --- | --- | --- | --- |
| **Onset age (years)** | **HR (95%CI)** | ***P* value** | **HR (95%CI)** | ***P* value** |
| **New-onset advanced CKM syndrome** | | | | |
| **N** | 16183 | | 18383 | |
| **<45** | 17.94 (2.37-136.01) | 0.005 | 2.76 (1.15-6.62) | 0.023 |
| **45-54** | 2.14 (1.51-3.05) | <0.001 | 2.54 (1.97-3.27) | <0.001 |
| **55-64** | 1.90 (1.61-2.24) | <0.001 | 2.01 (1.78-2.27) | <0.001 |
| **≥65** | 1.20 (1.08-1.34) | 0.001 | 1.13 (1.05-1.22) | <0.001 |
| ***P* for interaction** | <0.001 | | <0.001 | |
| **New-onset CKM syndrome stage 3** | | | | |
| **N** | 10969 | | 11763 | |
| **<45** | 14.03 (1.16-169.92) | 0.038 | 2.97 (0.79-11.12) | 0.108 |
| **45-54** | 2.08 (1.21-3.58) | 0.008 | 2.88 (1.89-4.38) | <0.001 |
| **55-64** | 1.61 (1.29-2.03) | <0.001 | 1.91 (1.60-2.28) | <0.001 |
| **≥65** | 1.12 (0.99-1.27) | 0.076 | 0.99 (0.90-1.07) | 0.803 |
| ***P* for interaction** | <0.001 | | <0.001 | |
| **New-onset CKM syndrome stage 4** | | | | |
| **N** | 7466 | | 8482 | |
| **<45** | 13.75 (1.81-104.54) | 0.011 | 3.32 (0.90-12.28) | 0.073 |
| **45-54** | 2.53 (1.62-3.95) | <0.001 | 2.67 (1.97-3.61) | <0.001 |
| **55-64** | 2.21 (1.76-2.76) | <0.001 | 2.11 (1.80-2.49) | <0.001 |
| **≥65** | 1.68 (1.47-1.92) | <0.001 | 1.69 (1.54-1.87) | <0.001 |
| ***P* for interaction** | 0.001 | | 0.027 | |
| **New-onset CKM syndrome stage 4a** | | | | |
| **N** | 7011 | | 7989 | |
| **<45** | 9.85 (1.24-78.09) | 0.030 | 3.16 (0.84-11.89) | 0.089 |
| **45-54** | 2.36 (1.48-3.77) | <0.001 | 2.41 (1.77-3.29) | <0.001 |
| **55-64** | 1.84 (1.47-2.30) | <0.001 | 2.01 (1.70-2.39) | <0.001 |
| **≥65** | 1.57 (1.36-1.80) | <0.001 | 1.60 (1.45-1.77) | <0.001 |
| ***P* for interaction** | 0.010 | | 0.061 | |
| **New-onset CKM syndrome stage 4b** | | | | |
| **N** | 1097 | | 1215 | |
| **<45** | NA | NA | NA | NA |
| **45-54** | 4.96 (1.85-13.32) | 0.002 | 3.82 (0.96-15.21) | 0.058 |
| **55-64** | 2.89 (1.94-4.29) | <0.001 | 3.41 (2.01-5.79) | <0.001 |
| **≥65** | 2.27 (1.86-2.76) | <0.001 | 2.21 (1.69-2.88) | <0.001 |
| ***P* for interaction** | 0.086 | | 0.134 | |

Note: The model was adjusted for smoking status, drinking status, physical activity, education, work type, and high-sensitivity C-reactive protein.

N, number of participants, including case subjects and control subjects; *P* for interaction, the interaction between cases and their ages at diagnosis.

**Table S19. The hazard ratios (95% CI) for all-cause mortality among participants with new-onset cases versus control subjects across age-groups: Sensitivity analysis excluding participants with follow-up less than 1 year,** **excluding participants who take regular physical activity, excluding participants with medication history at baseline.**

|  | **Excluding participants with follow-up less than 1 year** | | **Excluding participants who take regular physical activity** | | **Excluding participants with medication history** | |
| --- | --- | --- | --- | --- | --- | --- |
| **Onset age (years)** | **HR (95%CI)** | ***P* value** | **HR (95%CI)** | ***P* value** | **HR (95%CI)** | ***P* value** |
| **New-onset advanced CKM syndrome** | | | | | | |
| **N** | 34223 | | 28055 | | 28952 | |
| **<45** | 4.04 (1.64-9.96) | 0.002 | 4.09 (1.65-10.12) | 0.002 | 3.27 (1.29-8.26) | 0.012 |
| **45-54** | 2.49 (1.96-3.18) | <0.001 | 2.27 (1.77-2.92) | <0.001 | 2.37 (1.82-3.11) | <0.001 |
| **55-64** | 1.91 (1.70-2.15) | <0.001 | 1.97 (1.73-2.23) | <0.001 | 1.80 (1.58-2.04) | <0.001 |
| **≥65** | 1.19 (1.11-1.28) | <0.001 | 1.18 (1.09-1.27) | <0.001 | 1.14 (1.06-1.23) | <0.001 |
| ***P* for interaction** | <0.001 | | <0.001 | | <0.001 | |
| **New-onset CKM syndrome stage 3** | | | | | | |
| **N** | 22506 | | 17541 | | 19859 | |
| **<45** | 3.96 (1.10-14.30) | 0.036 | 5.94 (1.29-27.34) | 0.022 | 3.32 (1.24-12.64) | 0.029 |
| **45-54** | 3.20 (2.11-4.85) | <0.001 | 2.96 (1.93-4.53) | <0.001 | 2.64 (1.72-4.05) | <0.001 |
| **55-64** | 1.76 (1.49-2.07) | <0.001 | 1.77 (1.48-2.12) | <0.001 | 1.69 (1.41-2.02) | <0.001 |
| **≥65** | 1.07 (0.99-1.15) | 0.072 | 1.05 (0.97-1.15) | 0.241 | 1.02 (0.94-1.11) | 0.695 |
| ***P* for interaction** | <0.001 | | <0.001 | | <0.001 | |
| **New-onset CKM syndrome stage 4** | | | | | | |
| **N** | 15723 | | 12083 | | 12186 | |
| **<45** | 6.64 (1.50-29.40) | 0.012 | 4.18 (1.18-14.80) | 0.026 | 5.75 (1.24-26.71) | 0.026 |
| **45-54** | 2.57 (1.91-3.45) | <0.001 | 2.55 (1.87-3.46) | <0.001 | 2.58 (1.83-3.62) | <0.001 |
| **55-64** | 2.02 (1.73-2.36) | <0.001 | 2.12 (1.79-2.52) | <0.001 | 1.91 (1.60-2.28) | <0.001 |
| **≥65** | 1.71 (1.56-1.88) | <0.001 | 1.73 (1.56-1.91) | <0.001 | 1.79 (1.61-1.99) | <0.001 |
| ***P* for interaction** | <0.001 | | <0.001 | | 0.010 | |
| **New-onset CKM syndrome stage 4a** | | | | | | |
| **N** | 14805 | | 11379 | | 11529 | |
| **<45** | 5.81 (1.29-26.21) | 0.021 | 3.94 (1.09-14.23) | 0.037 | 5.63 (1.19-26.70) | 0.030 |
| **45-54** | 2.28 (1.69-3.09) | <0.001 | 2.25 (1.64-3.09) | <0.001 | 2.23 (1.57-3.15) | <0.001 |
| **55-64** | 1.90 (1.61-2.23) | <0.001 | 1.99 (1.67-2.38) | <0.001 | 1.87 (1.56-2.25) | <0.001 |
| **≥65** | 1.61 (1.46-1.78) | <0.001 | 1.66 (1.49-1.85) | <0.001 | 1.71 (1.53-1.91) | <0.001 |
| ***P* for interaction** | 0.002 | | 0.006 | | <0.001 | |
| **New-onset CKM syndrome stage 4b** | | | | | | |
| **N** | 2245 | | 1787 | | 1681 | |
| **<45** | 6.15 (0.62-60.66) | 0.120 | 6.49 (0.68-62.09) | 0.174 | 5.49 (0.59-66.29) | 0.129 |
| **45-54** | 4.84 (2.10-11.17) | 0.002 | 6.63 (2.51-17.54) | <0.001 | 3.83 (1.47-9.96) | 0.006 |
| **55-64** | 3.07 (2.90-4.50) | <0.001 | 3.78 (2.47-5.77) | <0.001 | 2.87 (1.86-4.42) | <0.001 |
| **≥65** | 2.20 (1.82-2.66) | <0.001 | 2.28 (1.87-2.78) | <0.001 | 2.32 (1.89-2.85) | <0.001 |
| ***P* for interaction** | 0.005 | | 0.002 | | 0.044 | |

Note: The model was adjusted for smoking status, drinking status, physical activity, education, work type, and high-sensitivity C-reactive protein.

N, number of participants, including case subjects and control subjects; *P* for interaction, the interaction between cases and their ages at diagnosis.

**Table S20. Subgroup analysis (based on sex) of the hazard ratios (95% CI) for all-cause mortality among participants with new-onset cases versus control subjects across age-groups.**

|  | **Male** | | **Female** | |
| --- | --- | --- | --- | --- |
| **Onset age (years)** | **HR (95%CI)** | ***P* value** | **HR (95%CI)** | ***P* value** |
| **New-onset advanced CKM syndrome** | | | | |
| **N** | 30902 | | 3664 | |
| **<45** | 4.71 (1.78-12.47) | 0.002 | 0.95 (0.13-7.11) | 0.959 |
| **45-54** | 2.38 (1.87-3.03) | <0.001 | 5.18 (1.48-18.12) | 0.010 |
| **55-64** | 1.91 (1.70-2.14) | <0.001 | 2.71 (1.74-4.20) | <0.001 |
| **≥65** | 1.12 (1.04-1.20) | 0.002 | 1.66 (1.31-2.11) | <0.001 |
| ***P* for interaction** | <0.001 | | 0.051 | |
| **New-onset CKM syndrome stage 3** | | | | |
| **N** | 21206 | | 1526 | |
| **<45** | 5.99 (1.29-27.79) | 0.022 | 1.71 (0.01-251.56) | 0.832 |
| **45-54** | 2.92 (1.95-4.37) | <0.001 | 3.12 (0.52-18.70) | 0.213 |
| **55-64** | 1.72 (1.46-2.02) | <0.001 | 5.41 (1.96-14.92) | 0.001 |
| **≥65** | 1.00 (0.93-1.08) | 0.3407 | 1.43 (1.02-2.02) | 0.038 |
| ***P* for interaction** | <0.001 | | 0.154 | |
| **New-onset CKM syndrome stage 4** | | | | |
| **N** | 13697 | | 2251 | |
| **<45** | 6.52 (1.47-28.91) | 0.014 | 1.20 (0.05-30.64) | 0.910 |
| **45-54** | 2.54 (1.89-3.41) | <0.001 | 14.25 (3.77-54.89) | 0.013 |
| **55-64** | 2.06 (1.76-2.41) | <0.001 | 2.40 (1.45-3.98) | <0.001 |
| **≥65** | 1.74 (1.58-1.91) | <0.001 | 1.66 (1.22-2.26) | <0.001 |
| ***P* for interaction** | <0.001 | | 0.075 | |
| **New-onset CKM syndrome stage 4a** | | | | |
| **N** | 12910 | | 2090 | |
| **<45** | 6.00 (1.32-27.35) | 0.021 | 1.20 (0.05-31.00) | 0.914 |
| **45-54** | 2.34 (1.72-3.17) | <0.001 | 4.61 (0.93-22.90) | 0.062 |
| **55-64** | 1.91 (1.62-2.25) | <0.001 | 2.10 (1.22-3.61) | 0.008 |
| **≥65** | 1.64 (1.49-1.82) | <0.001 | 1.62 (1.16-2.27) | 0.005 |
| ***P* for interaction** | 0.002 | | 0.336 | |
| **New-onset CKM syndrome stage 4b** | | | | |
| **N** | 1944 | | 368 | |
| **<45** | NA | NA | NA | NA |
| **45-54** | 4.16 (1.65-10.47) | 0.003 | NA | NA |
| **55-64** | 3.32 (2.23-4.94) | <0.001 | 3.53 (1.14-10.97) | 0.029 |
| **≥65** | 2.20 (1.82-2.66) | <0.001 | 3.05 (1.63-5.72) | <0.001 |
| ***P* for interaction** | 0.005 | | 0.219 | |

Note: The model was adjusted for smoking status, drinking status, physical activity, education, work type, and high-sensitivity C-reactive protein.

N, number of participants, including case subjects and control subjects; *P* for interaction, the interaction between cases and their ages at diagnosis.

**Table S21. Subgroup analysis (based on eGFR) of the hazard ratios (95% CI) for all-cause mortality among participants with new-onset cases versus control subjects across age-groups.**

|  | **Participants with eGFR <60 mL/min/1.73m^2^** | | **Participants with eGFR≥60 mL/min/1.73m^2^** | |
| --- | --- | --- | --- | --- |
| **Onset age (years)** | **HR (95%CI)** | ***P* value** | **HR (95%CI)** | ***P* value** |
| **New-onset advanced CKM syndrome** | | | | |
| **N** | 4663 | | 29903 | |
| **<45** | NA | NA | 6.33 (2.50-16.05) | <0.001 |
| **45-54** | 3.33 (0.80-13.86) | 0.098 | 2.35 (1.84-3.02) | <0.001 |
| **55-64** | 2.44 (1.60-3.74) | <0.001 | 1.86 (1.65-2.10) | <0.001 |
| **≥65** | 1.21 (1.03-1.43) | 0.019 | 1.12 (1.04-1.21) | 0.003 |
| ***P* for interaction** | 0.002 | | <0.001 | |
| **New-onset CKM syndrome stage 3** | | | | |
| **N** | 3871 | | 18861 | |
| **<45** | NA | NA | 14.87 (3.38-54.85) | <0.001 |
| **45-54** | NA | NA | 2.53 (1.60-4.00) | <0.001 |
| **55-64** | 2.46 (1.35-4.47) | 0.003 | 1.56 (1.31-1.86) | <0.001 |
| **≥65** | 1.17 (0.97-1.41) | 0.096 | 0.98 (0.90-1.07) | 0.611 |
| ***P* for interaction** | 0.070 | | <0.001 | |
| **New-onset CKM syndrome stage 4** | | | | |
| **N** | 1516 | | 14432 | |
| **<45** | NA | NA | 3.98 (1.12-14.13) | 0.033 |
| **45-54** | 3.48 (0.99-12.32) | 0.053 | 2.56 (1.90-3.46) | <0.001 |
| **55-64** | 1.77 (1.10-2.85) | 0.019 | 2.10 (1.79-2.46) | <0.001 |
| **≥65** | 1.35 (1.11-1.64) | 0.002 | 1.84 (1.66-2.04) | <0.001 |
| ***P* for interaction** | 0.009 | | 0.005 | |
| **New-onset CKM syndrome stage 4a** | | | | |
| **N** | 1208 | | 13792 | |
| **<45** | NA | NA | 4.21 (1.18-14.96) | 0.027 |
| **45-54** | 2.12 (0.58-7.79) | 0.256 | 2.40 (1.77-3.27) | <0.001 |
| **55-64** | 1.04 (0.60-1.81) | 0.884 | 2.03 (1.73-2.40) | <0.001 |
| **≥65** | 1.25 (1.01-1.55) | 0.044 | 1.75 (1.57-1.94) | <0.001 |
| ***P* for interaction** | 0.718 | | 0.007 | |
| **New-onset CKM syndrome stage 4b** | | | | |
| **N** | 767 | | 1545 | |
| **<45** | NA | NA | NA | NA |
| **45-54** | NA | NA | 5.41 (2.04-14.33) | <0.001 |
| **55-64** | 2.35 (0.93-5.97) | 0.072 | 3.76 (2.44-5.79) | <0.001 |
| **≥65** | 1.65 (1.17-2.34) | 0.005 | 2.53 (1.96-3.26) | <0.001 |
| ***P* for interaction** | 0.131 | | 0.023 | |

Note: The model was adjusted for smoking status, drinking status, physical activity, education, work type, and high-sensitivity C-reactive protein.

N, number of participants, including case subjects and control subjects; *P* for interaction, the interaction between cases and their ages at diagnosis.
